# Exosomal CTCF Confers Cisplatin Resistance in Osteosarcoma by Promoting Autophagy *via* the IGF2-AS/miR-579-3p/MSH6 Axis

**DOI:** 10.1155/2022/9390611

**Published:** 2022-05-31

**Authors:** Haibo Zhan, Jun Xiao, Po Wang, Fengbo Mo, Kaihuang Li, Fengfen Guo, Xiaolong Yu, Xuqiang Liu, Bin Zhang, Min Dai, Hucheng Liu

**Affiliations:** ^1^Department of Orthopedics, The First Affiliated Hospital of Nanchang University, Nanchang, 330006 Jiangxi, China; ^2^Artificial Joints Engineering and Technology Research Center of Jiangxi Province, Nanchang, 330006 Jiangxi, China; ^3^Medical Innovation Center, The First Affiliated Hospital of Nanchang University, Nanchang, 330006 Jiangxi, China

## Abstract

Cancer-derived exosomes participate in carcinogenesis and progression of cancers, including metastasis and drug-resistance. Of note, CTCF has been suggested to induce drug resistance in various cancers. Herein, we aim to investigate the role of cisplatin- (CDDP-) resistant osteosarcoma- (OS-) derived exosomal CTCF in OS cell resistance to CDDP and its mechanistic basis. Differentially expressed transcription factors, long noncoding RNAs (lncRNAs), miRNAs, and genes in OS were retrieved using bioinformatics approaches. Exosomes were extracted from CDDP-resistant OS cells and then cocultured with parental OS cells, followed by lentiviral transduction to manipulate the expression of CTCF, IGF2-AS, miR-579-3p, and MSH6. We assessed the *in vitro* and *in vivo* effects on malignant phenotypes, autophagy, CDDP sensitivity, and tumor formation of OS cells. It was established that CTCF and IGF2-AS were highly expressed in CDDP-resistant OS cells, and the CDDP-resistant OS cell-derived exosomal CTCF enhanced IGF2-AS transcription. CDDP-resistant OS-derived exosomes transmitted CTCF to OS cells and increased CDDP resistance in OS cells by activating an autophagy-dependent pathway. Mechanistically, CTCF activated IGF2-AS transcription and IGF2-AS competitively bound to miR-579-3p to upregulate MSH6 expression. Additionally, the promoting function of exosomal CTCF-mediated IGF2-AS/miR-579-3p/MSH6 in OS cell resistance to CDDP was confirmed *in vivo*. Taken together, CDDP-resistant OS-derived exosomal CTCF enhanced resistance of OS cells to CDDP via activating the autophagy-dependent pathway, providing a potential therapeutic consideration for OS treatment.

## 1. Introduction

Osteosarcoma (OS) is the most common primary malignant bone tumor, mainly occurring in children and adolescents [1]. Tall stature, high birth weight, certain inherited cancer susceptibility syndromes, and common genetic variants are the main risk factors for OS [2]. Surgery combined with chemotherapy has greatly improved the prognosis of patients with OS, but the prognosis of recurrent or metastatic OS is a big challenge [3]. Cisplatin (CDDP) is regarded as a standard drug for the treatment of OS, but the resistance of OS to CDDP limits its effectiveness [4]. OS is a tumor believed to originate from bone-forming mesenchymal stem cells, and abnormal oncogene activation and tumor suppressor gene inactivation due to somatic mutations and epigenetic mechanisms play a key pathogenic role in OS [5]. In this regard, efforts are required to explore mutated epigenetic mechanisms involved in OS progression and OS resistance to CDDP for the development of OS treatment.

Exosomes are cell-derived extracellular vesicles which participate in intercellular communication and induce changes in cell behavior by transporting proteins, DNA, RNA, or lipids between cells and are concerned with tumorigenesis and drug resistance of OS [6]. CCCTC-binding factor (CTCF) is a highly conserved zinc finger protein that regulates high-order chromatin tissue and participates in various gene regulatory processes, which is often altered by hemizygous deletions or mutations in human cancers [7, 8]. Although CTCF is essential for cell survival, inadequate haploid CTCF is related to tumor development and hypermethylation [9]. CTCF has been revealed as a critical transcriptional factor to mediate long noncoding RNAs (lncRNAs) to affect cancer progression [10, 11]. Long noncoding RNA IGF2-AS is upregulated in gastric adenocarcinoma tissues and is associated with poor prognosis in gastric adenocarcinoma patients [12]. A previous report has pointed out the elevated IGF2 expression in OS after chemotherapy, which prompted us to examine its functional contribution to drug resistance [13]. It was generally known that lncRNAs modulate the expression of different microRNAs (miRNAs), and the binding of miRNAs to lncRNAs led to the increase in the expression of miRNA target genes [14]. Using bioinformatics software, we found that IGF2-AS could competitively bind to microRNA-579-3p (miR-579-3p) to upregulate MutS homolog 6 (MSH6) expression. miR-579-3p is strongly downregulated before and after resistance to targeted therapies in matched tumor samples from patients [15]. Further, it has been reported that miR-579-3p inhibits proliferation, invasion, and migration of squamous cell lung cancer cells [16]. MSH6 is one of the members of the MutS family which could heterodimerize with MSH2 to form a mismatch recognition complex to mediate DNA mismatch repair (MMR) in eukaryotes [17]. Silencing MSH6 combined with CDDP inhibited OS cell proliferation [18].

Given the aforementioned evidence, we proposed a hypothesis that exosomes delivering CTCF could regulate the IGF2-AS/miR-579-3p/MSH6 axis to enhance resistance of OS cells to CDDP.

## 2. Materials and Methods

### 2.1. Ethics Statement

The experiments involving animals were performed under the approval of the Animal Ethics Committee of the First Affiliated Hospital of Nanchang University and in compliance with the recommendations in the *Guide for the Care and Use of Laboratory Animals* of the National Institutes of Health.

### 2.2. In Silico Prediction

The hTFtarget database was used to predict the upstream transcription factor of IGF2-AS. The promoter sequence of IGF2-AS was obtained from the UCSC database. The JASPAR database was applied to predict the binding sites of transcription factors and downstream promoters. The Vesiclepedia database was adopted to retrieve the enrichment of transcription factors in exosomes.

The Gene Expression Omnibus (GEO) database was used to obtain the OS-related GSE41445 expression profile. There were 57 samples in the GSE41445 expression profile, which were not related to this study. After deletion of these 57 samples, 3 normal samples and 3 OS samples were left. The limma package in R language was used for differential analysis. Top 300 genes with logFC > 1 were considered significantly highly expressed genes.

The GeneCards database was used to identify genes related to OS. Related genes were retrieved through the STRING database, followed by construction of an interaction network. The Cytoscape database was employed for drawing and calculating the core level. KEGG enrichment analysis was conducted through the KOBAS 3.0 database. The MEM database was applied to perform large-scale analysis to determine the coexpression relationship among genes, and the lncATLAS database was used to predict the subcellular location of lncRNA to determine its downstream regulatory mechanism. The StarBase database was adopted to predict the relationship of lncRNA-miRNA-gene and obtain binding sites.

### 2.3. Cell Culture and Lentiviral Transduction

OS cells U-2OS (SCSP-5030) and MG63 (TCHu124) were purchased from the Cell Bank of the Chinese Academy of Sciences (Shanghai, China). The CDDP-resistant cell lines MG63/CDDP and U-2OS/CDDP were constructed in our laboratory and cultured in Roswell Park Memorial Institute- (RPMI-) 1640 medium (PM150110, Procell Co., Ltd., Wuhan, China) containing 10% fetal bovine serum (FBS, 164210), 100 U/mL penicillin, and 100 U/mL streptomycin (PB180120). Human osteoblasts (hFOB) and human embryonic kidney 293T (HEK-293 T) cells were purchased from the Shanghai Institute of Biochemistry and Cell Biology (Shanghai, China) and cultured in Dulbecco's modified Eagle medium (DMEM; PM150210, Procell, Wuhan, China) containing 10% FBS, 100 U/mL penicillin, and 100 U/mL streptomycin. Cells were placed and cultured in a cell incubator at 37°C with 5% CO_2_.

To study the transduction efficiency of short hairpin RNA (shRNA) target IFG2-AS (sh-IFG2-AS), MG63 cells were transduced with lentiviral sh-IGF2-AS-1 (5′-CGGCCTGGGAAGTAGGACTAA-3′), sh-IGF2-AS-2 (5′-TACAAACCCAGCTCCTTTCTC-3′), and their negative control (sh-NC; 5′-TTCTCCGAACGTGTCACGT-3′). To study the transduction efficiency of sh-MSH6, MG63/CDDP cells were transduced with lentiviral sh-MSH6-1 (5′-GCACAACTTACGTAACAGA-3′), sh-MSH6-2 (5′-GGCTGTAAACGATACTGGA-3′), and their NC (5′-TTCTCCGAACGTGTCACGT-3′). Lentiviruses were purchased from Sangon Biotech Co., Ltd. (Shanghai, China). shRNA sequences and lentiviruses were provided by Sangon Biotech Co., Ltd. All experimental steps were carried out according to the instruction manuals.

The effects of exosomes carrying CTCF (Exo-CTCF) on autophagy and CDDP resistance of CDDP-resistant OS cells via regulating IGF2-AS expression were examined. After transduction with lentivirus for 24 h, MG63 cells were treated with 10 *μ*g Exo-CTCF and 1 nM rapamycin (HY-10219, MCE) for 48 h. Cells were treated with Exo-CTCF alone (Exo-CTCF) or in the presence of lentiviral sh-NC (Exo-CTCF+sh-NC), lentiviral sh-IGF2-AS (Exo-CTCF+sh-IGF2-AS), or/and rapamycin (Exo-CTCF+sh-IGF2-AS+rapamycin). Those without any treatment functioned as the control. All above-mentioned cells were treated with 8 *μ*M CDDP (HY-17394, MCE) for 48 h and then collected for subsequent testing.

### 2.4. Construction of CDDP-Resistant Cell Lines

The CDDP-resistant cell lines MG63/CDDP and U-2OS/CDDP were derived from the parental cells MG63 and U-2OS. The parental cells MG63 and U-2OS were seeded in a cell culture dish. After cell confluence reached 60%-70%, cells were added with gradient CDDP (1, 2, 4, 8, 16, and 32 *μ*M) for 2 days. Cells were cultured with CDDP-free complete medium till the cells grew well. Upon cell confluence reaching 60%-70%, CDDP treatment was continued, and each concentration was repeated 6 times. After 9 months, the CDDP-resistant cell lines were successfully constructed.

### 2.5. Cell Counting Kit-8 (CCK-8) Assay

CCK-8 (C0037, Beyotime Biotechnology, Shanghai, China) was used to detect cell viability. The cells (MG63, U-2OS, MG63/CDDP, and U-2OS/CDDP) were seeded in a 96-well plate at a density of 2 × 10^4^ cells/well. Cells were treated with a specified dose of CDDP (0, 1, 2, 4, 8, 16, 32, 64, and 128 *μ*M) for 48 h, or MG63 cells were treated with 8 *μ*M CDDP and 1 nM rapamycin for 48 h. Then, 10 *μ*L of CCK-8 was added to each well. After incubating for 2 h in the cell incubator, the absorbance at 450 nm was measured on a microplate reader.

### 2.6. Western Blot Analysis

Total proteins from cells, tissues, and exosomes were extracted using phenylmethylsulfonyl fluoride- (PMSF-) containing radioimmunoprecipitation assay (RIPA) lysis buffer (P0013B, Beyotime Biotechnology, Shanghai, China). A bicinchoninic acid (BCA) protein assay kit (P0028, Beyotime Biotechnology, Shanghai, China) was used for protein concentration determination. Proteins were boiled at 100°C for 10 min to denature the protein and stored at -80°C for later use. According to the size of the target protein band, 8%-12% SDS electrophoresis gel was prepared. Proteins were separated by 10% sodium dodecyl sulfate (SDS) gel electrophoresis. After separation, proteins were transferred to a polyvinylidene fluoride (PVDF) membrane (1620177, Bio-Rad) and blocked with 5% bovine serum albumin (BSA) at room temperature for 1 h. The membrane was probed with primary antibodies to CTCF (ab128873, 1 : 5000, rabbit), MSH6 (ab208940, 1 : 1000, rabbit), p62 (ab109012, 1 : 10000, rabbit), Beclin1 (Ab210498, 1 : 1000, rabbit), and LC3 (ab192890, 1 : 2000, rabbit) overnight at 4°C. The next day, the membrane was washed with 1× Tris buffered saline with Tween (TBST) at room temperature. The protein was preprobed with a horseradish peroxidase- (HRP-) labeled goat anti-rabbit immunoglobulin G (IgG) antibody (ab6721, 1 : 5000) as a secondary antibody for 1 h at room temperature. All above-mentioned antibodies were obtained from Abcam Inc. (Cambridge, UK). The membrane was immersed in the electrogenerated chemiluminescence (ECL) reaction solution (P0018S, Beyotime Biotechnology, Shanghai, China) at room temperature for 1 min. Images were captured on the Image Quant LAS 4000C gel imager (General Electric Company, NY, USA). Glyceraldehyde-3-phosphate dehydrogenase (GAPDH; A01021, 1 : 5000, rabbit, Abbkine, USA) was used as the internal reference. The gray value ratio of the target band to the internal reference band was used as the relative expression of the protein to detect the expression of each protein.

### 2.7. Reverse Transcription-Quantitative Polymerase Chain Reaction (RT-qPCR)

Total RNA of tissues or cells was extracted in strict accordance with the instructions of the TRIZOL kit (15596-018, Thermo Fisher Scientific, Waltham, MA, USA). The purity and concentration of obtained RNA were evaluated by measuring the absorbance of the solution at 260 and 280 nm by spectrophotometry. The A260/A280 ratio of sample was 1.8-2.0. For miRNA, the polyA tailing method was used to generate cDNA of the miRNA containing the polyA tail based on the detection kit (containing universal PCR primer R and U6 universal PCR primer R; B532451, Sangon Biotech, Co., Ltd., Shanghai, China). The mRNA was reversely transcribed according to the instructions of the cDNA reverse transcription kit (RR047A, Takara, Tokyo, Japan). The reverse-transcribed cDNA was diluted to 50 ng/*μ*L. PCR was performed according to the instructions of LightCycler 480 SYBR Green I Master (04707516001, Roche, Germany). The gene expression was quantified by the 2^−ΔΔCt^ method, with U6 as the miRNA internal reference and GAPDH as the internal reference for other genes. The primer sequences are shown in Supplementary Table 1.

### 2.8. Isolation and Identification of Exosomes

Differential centrifugation was used to separate exosomes from the cell culture supernatant of MG63 and MG63/CDDP. The collected supernatant was centrifuged at 300 × *g* for 10 min, 2000 × *g* for 10 min, and 10000 × *g* for 30 min. Exosomes were resuspended with phosphate PBS and filtered with a 0.22 *μ*m filter to remove smaller cell debris. After being washed with PBS, exosomes were resuspended by ultracentrifugation at 110000 × *g* for 2 h to remove PBS. The supernatant was subjected to ultracentrifugation at 110000 × *g* for 2 h. The obtained precipitates were exosomes. The collected exosomes were resuspended in PBS and filtered with 0.22 *μ*m to remove smaller cell debris. Exosomes were resuspended with PBS and ultracentrifuged at 110000 × *g* for 2 h to remove the PBS. Then, the second ultracentrifugation under the same conditions was performed. The precipitate was collected and stored at -80°C for later use. The above centrifugation was performed at 4°C. Exosomes were cultured at cell culture medium containing exosome-free serum (C38010050, Viva Cell, Shanghai, China).

Transmission electron microscopy (H-7650, HITACHI, Tokyo, Japan) was applied to identify exosomes. Total 10 *μ*L of exosomes was dropped on the parafilm, and the copper mesh Formvar film was placed face down on the suspension. Each exosome sample was prepared with 2-3 copper meshes. Then, 100 *μ*L PBS was added to the parafilm, and the copper mesh (Formvar membrane facing down) was placed on a PBS droplet with tweezers for 5 min. The copper mesh was treated with 50 *μ*L of 1% glutaraldehyde droplets for 5 min and treated in 100 *μ*L of double distilled water for 2 min. The copper mesh was treated with a 50 *μ*L drop of uranyl oxalate for 5 min. The copper mesh was then placed on a 50 *μ*L drop of methylcellulose-UA for 10 min. The copper mesh was removed with a stainless steel ring, followed by gently absorbing the excess liquid on the filter paper, leaving a thin layer of methyl cellulose membrane. The dried copper mesh was placed under 100 keV to take electron micrographs.

Identification of exosome surface marker proteins was determined by Western blot analysis. Exosomes were dissolved in RIPA buffer containing MSF and quantitatively identified by using the BCA protein analysis kit. Western blot analysis was performed with antibodies as follows: CD9 (ab92726, 1 : 2000, rabbit, Abcam Inc.), CD63 (ab134045, 1 : 1000, rabbit, Abcam Inc.), and calnexin (ab133615, 1 : 2000, rabbit, Abcam Inc.).

Exosome size was evaluated by nanoparticle tracking analysis (NTA), which could automatically track and determine particle size based on the diffusion coefficient of the Brownian motion. Exosomes were resuspended and mixed in 1 mL PBS, and the filtered PBS was used as a control. Then, the diluted exosomes were injected into the NanoSight LM10 instrument to measure the particle size.

### 2.9. Uptake of Exosomes

An equal volume of 2 *μ*M PHK67 dye (MINI67, Sigma-Aldrich, St. Louis, MO, USA) was added to the extracted exosomes. After 5 min of culture at room temperature, 10 mL of exosome medium was added into exosomes to stop staining. Exosomes were centrifuged at 400 × *g* for 10 min, and the supernatant was discarded. Then, 10 mL of exosomes was added and centrifuged again at 400 × *g* for 5 min. The supernatant was discarded, and the precipitate was obtained after which was PHK67-labeled exosomes. A special cell slide was placed on the top of the petri dish, and MG63 cells were cultured in the petri dish. When cell density reached 50%, cells were added to exosomes labeled with PHK67 and coincubated at room temperature for 24 h. The slide was taken out and washed slowly with PBS 3 times. Cells were soaked in 4% paraformaldehyde at room temperature for 30 min, permeabilized with 2% Triton X-100 for 15 min, and blocked with 2% BSA for 45 min. The blocking solution was discarded. After staining with DAPI (2 *μ*g/mL, C1005, Beyotime, Shanghai, China), the slides were mounted. Fluorescence intensity was detected by using an upright fluorescence microscope.

### 2.10. Chromatin Immunoprecipitation (ChIP)

To detect the enrichment of CTCF in the promoter region of IGF2-AS, the EZ-Magna ChIP TMA kit (Merck Millipore, Billerica, MA) was used for ChIP experiments. The OS cells in the logarithmic growth phase were cross-linked and cultured with 1% formaldehyde for 10 min. The cells were treated with the protease inhibitor and then sonicated until 200-1000 bp chromatin fragments were obtained. In each group, 100 *μ*L of supernatant (DNA fragment) was added to 900 *μ*L ChIP dilution buffer, 20 *μ*L of 50 × PIC, and 60 *μ*L Protein A Agarose/Salmon Sperm DN to invert and mix at 4°C for 1 h. After centrifugation, the supernatant was taken out, and 20 *μ*L was used as input. In the experimental group, the supernatant was mixed with 2 *μ*g of CTCF antibody (ab128873, rabbit, Abcam Inc.). In the NC group, 2 *μ*g of anti-IgG (ab172730, rabbit, Abcam Inc.) was added, and 60 *μ*L of Protein A Agarose/Salmon was added to each tube. Sperm DNA was inverted at 4°C for 2 h. After centrifugation, the precipitate was washed with 1 mL of low-salt buffer, high-salt buffer, LiCl solution, and TE, respectively. Each tube was eluted twice with 250 mL ChIP Wash Buffer. Then, 20 mL of 5 M NaCl was used to reverse cross-link. The DNA was recovered, and the promoter sequence of IGF2-AS in the complex (F: 5′-AGAATTCAGGGGCCCCATCC-3′, R: 5′-CTGCATCTGCACTCAGACGG-3′) was conducted with quantification.

### 2.11. Dual-Luciferase Reporter Gene Assay

The IGF2-AS sequence containing the CTCF binding site and mutation binding site was cloned into a pGL3-Promoter luciferase reporter vector (E1751, Promega, Madison, WI, USA) to construct IGF2-AS-wild type (WT) and IGF2-AS-mutant type (MUT) reporter vectors. HEK-293T cells were cotransfected with oe-NC or oe-CTCF and the above-mentioned reporter vectors using the Lipofectamine 2000 kit (11668019, Invitrogen, Carlsbad, CA). After 24 h of transfection, the supernatant of HEK-293T cells was collected. The luciferase activity was measured using the dual-luciferase reporter analysis system (E1910, Promega, Madison, WI, USA). The luciferase activity was directly measured by the ratio of firefly luciferase to Renilla luciferase. The same method was used to detect the binding ability of IGF2-AS with miR-579-3p and miR-579-3p with MSH6.

### 2.12. RNA Immunoprecipitation (RIP)

The RIP assay was performed according to the instructions of the EZ-Magna RIP kit (17-701, Millipore, Billerica, MA, USA). Cells were lysed with RIP lysis buffer. After resuspending magnetic beads in RIP Wash Buffer, cells were probed with the AGO2 antibody (ab186733, 1 : 30, Abcam Inc.) or anti-rabbit IgG (ab6721, 1 : 30, Abcam Inc.) for 30 min. Cells were incubated with the lysed cell supernatant at room temperature overnight at 4°C. After a short centrifugation, the supernatant was discarded. Cells were added with RIP Wash Buffer and washed 6 times. The RNA in the magnetic bead-antibody complex was extracted. The expression of IGF2-AS and miR-579-3p in the complex was analyzed by RT-qPCR. All experimental steps were performed according to the instruction manual.

### 2.13. Fluorescence In Situ Hybridization (FISH)

RiboTM lncRNA FISH Probe Mix (Red) (C10920, Ruibo Bio, China) was used to detect the subcellular colocalization of IGF2-AS and miR-579-3p. Cells were seeded in a 24-well plate. When confluence reached 60-70%, cells were fixed in 4% formaldehyde for 10 min. The membrane was ruptured with 0.5% TritonX-100 for 5 min. Cells in each well were added with 200 *μ*L of prehybridization solution and incubated at 37°C for 30 min, followed by incubating with Cy3-labeled IGF2-AS and FITC-labeled miR-579-3p cell probes, overnight at 37°C. The cells were stained with DAPI (C1005, Beyotime Biotechnology, Shanghai, China). Finally, the cells were observed and photographed in 5 different fields of view under a fluorescence microscope (Olympus, Tokyo, Japan).

### 2.14. Transmission Electron Microscope (TEM) for Autophagosome Observation

The OS cells were fixed with 2.5% glutaraldehyde solution (DF0156, Beijing Leagene Biotechnology, Beijing, China) at 4°C overnight. After cells were rinsed with PBS, 1% osmium tetroxide solution was added and fixed at room temperature for 1 h. The samples were dehydrated, put into the mixture of pure acetone, and embedded. The cultured samples were infiltrated, embedded, ultramicrosectioned, and stained (3% uranyl acetate-lead citrate double staining). Autophagosomes in the cells were observed under a TEM and counted.

### 2.15. Flow Cytometric Detection

Cells were collected and treated with trypsin without ethylenediaminetetraacetate (EDTA) to obtain a single cell suspension. Subsequently, the Annexin V-fluorescein isothiocyanate (FITC) apoptosis detection kit (KGA107, KeyGEN BioTECH Corp., Ltd., Jiangsu, China) was used to detect cell apoptosis. The collected cells were suspended with 500 *μ*L of binding buffer. After adding 5 *μ*L of Annexin V-FITC and 5 *μ*L of propidium iodide (PI), cells were reacted for 15 min at room temperature in the dark and tested by flow cytometry within 1 h.

### 2.16. Xenografts in Nude Mice

Six-week-old female BALB/c nude mice (401; Beijing Vital River Laboratory Animal Technology Co., Ltd., Beijing, China) were raised under nonpathogenic conditions at 26-28°C with 50-65% humidity. MG63 cells were transduced with lentiviral sh-NC and sh-MSH6 and resuspended in PBS. The collected transduced MG63 OS cells were counted, resuspended in PBS, and injected into the right armpit of nude mice (1 × 10^6^ cells, cells/mouse).

The nude mice were randomly inoculated with lentiviral sh-NC-transduced MG63 cells or lentiviral sh-MSH6-transduced MG63 cells under the armpit and injected with 100 *μ*L PBS or 10 *μ*g MG63/CDDP cell-derived exosomes via the tail vein (*n* = 6). All above mice were injected with CDDP. Exosomes were configured in sterile PBS and injected into the tail vein every 3 days (10 *μ*g exosomes in 100 *μ*L PBS was injected each time).

When the tumor grew to 50 mm^3^, nude mice were injected intraperitoneally with CDDP (3 mg/kg) twice a week. Tumor volume was measured with a Vernier caliper every 1 week. The tumor volume calculation formula was *W* = 1/2∗*a*∗*b*^2^ (*a* represented the long diameter, *b* represented the short diameter). After 4 weeks, the nude mice were sacrificed to remove the xenograft tumor tissues.

### 2.17. Statistical Analysis

Data statistical analyses were processed using SPSS 21.0 statistical software (IBM Corp. Armonk, NY). Measurement data were expressed as mean ± standard deviation. Data between two groups were compared using the paired *t*-test or unpaired *t*-test. Comparisons among multiple groups were conducted by one-way analysis of variance (ANOVA), followed by Tukey's post hoc test. Two-way analysis of variance was applied for cell viability comparison among multiple groups, and repeated measures analysis of variance for tumor data comparison. A value of *p* < 0.05 indicated significant difference.

## 3. Results

### 3.1. Exosomes Derived from OS Cells Deliver CTCF into OS Cells to Upregulate IGF2-AS Expression

**Figure 1:**
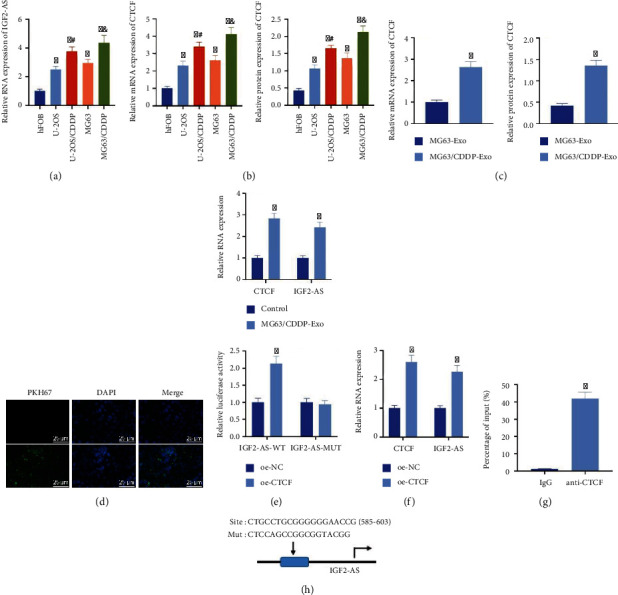
The upregulated expression of CTCF and IGF2-AS is identified in OS cells. (a) RT-qPCR detection of IGF2-AS expression in CDDP-sensitive and CDDP-resistant OS cells. (b) Detection of the expression of CTCF in human osteoblasts (hFOB) and OS cells (U-2OS and MG63) and their CDDP-resistant cell lines (MG63/CDDP and U-2OS/CDDP) by RT-qPCR and Western blot analysis (^∗^*p* < 0.05*vs.* hFOB, ^#^*p* < 0.05*vs.* U-2OS, and ^&^*p* < 0.05*vs.* MG63). (c) Detection of the expression of CTCF in MG63-Exo and MG63/CDDP-Exo by RT-qPCR and Western blot analysis. (d) Observation of the uptake of exosomes by MG63 cells under a confocal fluorescence microscope. (e) RT-qPCR detection for the expression of CTCF and IGF2-AS in MG63 cells cocultured with MG63/CDDP-Exo. (f) RT-qPCR applied to determine the expression of CTCF and IGF2-AS after oe-CTCF transduction in MG63 cells. (g) Validation of the binding of the transcription factor CTCF to the promoter region of IGF2-AS by ChIP. (h) Verification of the binding of CTCF and IGF2-AS through luciferase activity detection by the dual-luciferase reporter gene assay. ^∗^*p* < 0.05*vs.* MG63-Exo/control/oe-NC/IgG. Cell experiment was repeated three times.

In this study, we constructed CDDP-resistant OS cells. CCK-8 results showed that the IC50 values of CDDP-resistant cells MG63/CDDP (39.49 *μ*M) and U-2OS/CDDP (74.89 *μ*M) for CDDP were significantly higher than those of the corresponding parent cell MG63 (5.96 *μ*M) and U-2OS/CDDP cells (11.54 *μ*M) (Supplementary Figure 1), which indicated that the CDDP-resistant OS cells were successfully constructed. We found that IGF2-AS was significantly highly expressed in CDDP-resistant OS cells through RT-qPCR detection results (Figure 1(a)).

According to the prediction of the hTFtarget database, 70 upstream transcription factors of IGF2-AS were obtained. Among them, CTCF targeted IGF2-AS in 24 types of tissues (Supplementary Table 2), which was the transcription factor targeting the most types of tissues. This result indicated that CTCF might be the key upstream gene of IGF2-AS. Subsequently, an analysis through the Vesiclepedia database found that CTCF was enriched in tumor-derived exosomes. Therefore, we speculated that exosomes from tumor cells delivered CTCF to activate IGF2-AS.

Exosomes were isolated from the supernatant of MG63 and MG63/CDDP cell culture medium, and the morphology of exosomes was observed by TEM (Supplementary Figure 2A). The isolated exosomes had a typical exosome-like morphology, with basically uniform round or oval membranous vesicles. NTA showed that the diameter of exosomes ranged from 30 nm to 150 nm (Supplementary Figure 2B). Western blot results showed that the surface of exosomes significantly expressed the surface markers CD63 and CD9 of exosomes but did not express calnexin (Supplementary Figure 2C). The above results confirmed that extracted exosomes were successfully derived from OS cells.

In addition, the results of RT-qPCR and Western blot analysis showed that the expression of CTCF in CDDP-resistant OS cells was higher than that in CDDP-sensitive OS cells (Figure 1(b)). In addition, the expression of CTCF in MG63/CDDP-Exo was higher than that in MG63-Exo (Figure 1(c)). After cocultivating PKH67- (green) labeled MG63/CDDP-Exo with MG63 cells for 24 h, observation under a fluorescence microscope revealed that MG63 cells presented obvious PKH67 staining, which confirmed that MG63 cells had successfully taken up exosomes (Figure 1(d)). RT-qPCR results showed that the expression of CTCF and IGF2-AS after coculture with MG63/CDDP-Exo was higher than that in the control (Figure 1(e)).

Moreover, oe-CTCF was transduced in MG63 cells, and the RT-qPCR results showed that the expression of CTCF and IGF2-AS upon the oe-CTCF treatment was elevated (Figure 1(f)). ChIP experiment found that CTCF could directly bind to the promoter region of IGF2-AS (Figure 1(g)). The dual-luciferase reporter gene assay showed that the cell luminescence intensity was increased after overexpression of CTCF, but the cell luminescence intensity did not change significantly after the site mutation (Figure 1(h)).

The above results indicated that CTCF and IGF2-AS were highly expressed in CDDP-resistant OS cells, and the CDDP-resistant OS cell-derived exosomes carrying CTCF could promote the transcriptional expression of IGF2-AS in OS cells.

### 3.2. OS-Derived Exosomal CTCF Activates the Autophagy Signaling Pathway via Upregulating IGF2-AS to Enhance the CDDP Resistance of OS Cells

**Figure 2:**
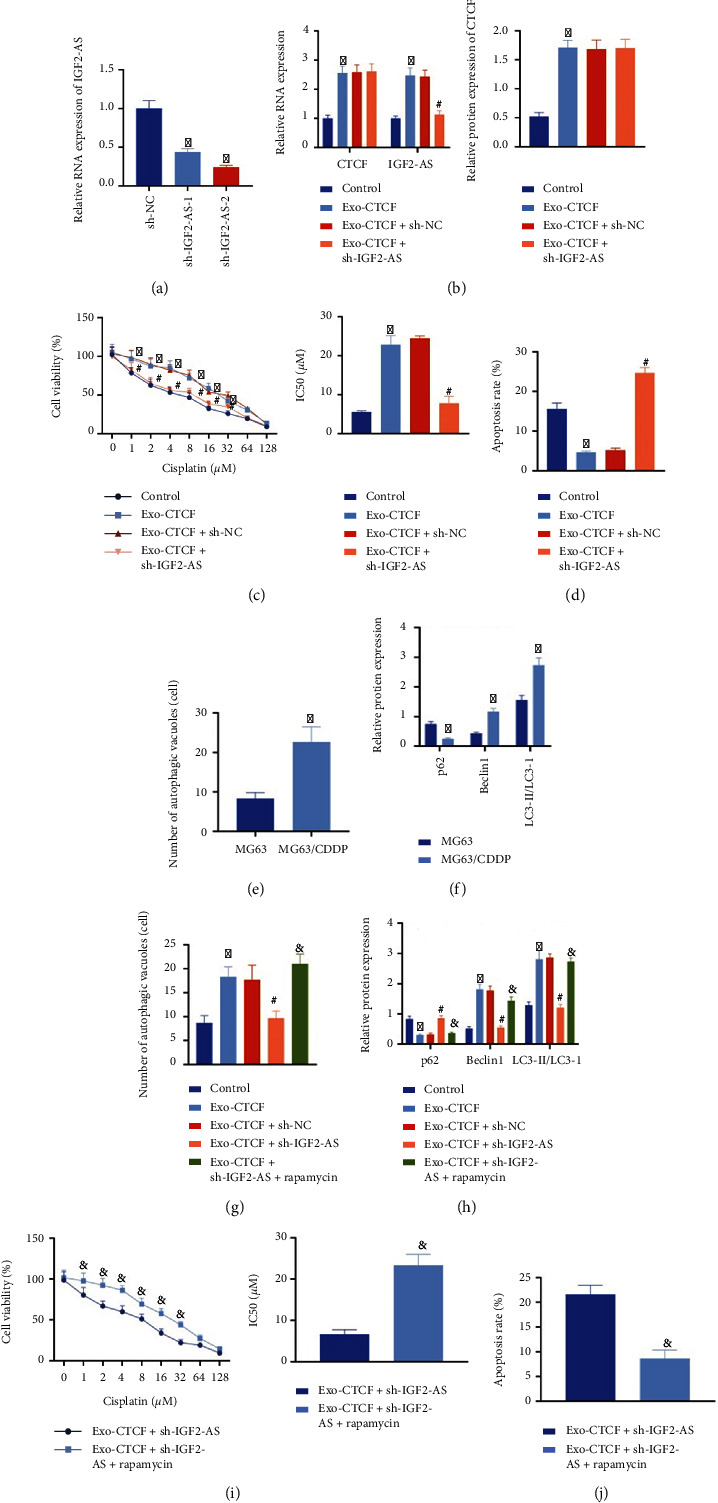
Exosomal CTCF-mediated IGF2-AS activates autophagy to enhance CDDP resistance of OS cells. (a) Detection of the silencing efficiency of lentiviral sh-IGF2-AS in MG63 cells by RT-qPCR. (b) Determination of the expression of CTCF and IGF2-AS in the parental cells by RT-qPCR and Western blot analysis. (c) CCK-8 applied to detect the IC50 value of OS cells to CDDP. (d) Flow cytometry adopted for detection of the apoptosis in OS cells. (e) TEM data of autophagy in MG63 and MG63/CDDP cells after the intervention of CDDP; the red arrow was the autophagosome. (f) Western blot analysis to measure the expression of autophagy-related genes p62, Beclin1, and LC3 in MG63 and MG63/CDDP cells after CDDP intervention. (g) Autophagy in the cells observed by TEM after the intervention of the autophagy agonist rapamycin. (h) The expression of autophagy-related genes p62, Beclin1, and LC3 in cells determined by Western blot analysis after the intervention of the autophagy agonist rapamycin. (i) CCK-8 used to examine the IC50 value of OS cells to CDDP after rapamycin intervention. (j) Flow cytometry to detect the apoptosis of OS cells after rapamycin intervention. ^∗^*p* < 0.05*vs.* sh-NC/control/MG63, ^#^*p* < 0.05*vs.* Exo-CTCF+sh-NC, and ^&^*p* < 0.05*vs.* Exo-CTCF+sh-IGF2-AS. Cell experiment was repeated three times.

We further moved to test the influence of exosomal CTCF on CDDP resistance and the involvement of IGF2-AS. RT-qPCR was applied to detect the silencing efficiency of lentiviral sh-IGF2-AS in MG63 cells. It was noted that lentiviral sh-IGF2-AS-1 and sh-IGF2-AS-2 could knock down the expression of IGF2-AS in MG63 cells, among which lentiviral sh-IGF2-AS-2 had better silencing effect and was used in subsequent experiments (Figure 2(a)).

Lentiviral sh-IGF2-AS was transduced in parental cells after coculture with exosomes derived from CDDP-resistant OS cells. We found that the expression of CTCF and IGF2-AS was elevated in the parental cells after coculture with Exo-CTCF. The upregulated expression of IGF2-AS caused by Exo-CTCF was reversed by additional sh-IGF2-AS treatment, but there was no significant difference in the expression of CTCF (Figure 2(b)).

Subsequently, MG63 cells were treated with different concentrations of CDDP. CCK-8 and flow cytometry results showed that the cell viability and IC50 were increased upon the coculture with Exo-CTCF, while cell apoptosis was diminished. Compared with the coculture with Exo-CTCF alone, the cell viability and IC50 value were diminished in cells with Exo-CTCF and sh-IGF2-AS treatment, and cell apoptosis was increased (Figures 2(c) and 2(d)). The above results indicated that exosomes derived from CDDP-resistant OS cells delivered CTCF to increase the expression of IGF2-AS, thereby enhancing the CDDP resistance of OS cells.

Ultrastructural analysis by TEM showed that, after 48 h of intervention with 8 *μ*M CDDP, the number of autophagosomes in MG63/CDDP cells was higher than that in MG63 cells (Figure 2(e)). Western blot analysis showed that compared with untreated MG63 cells, the expression of p62 in MG63/CDDP cells was reduced, and the expression of Beclin1 and the ratio of LC3-II/LC3-I were increased. This result indicated that the autophagy was increased in CDDP-resistant OS cells (Figure 2(f)).

MG63 cells were subjected to lentiviral transduction and autophagy agonist rapamycin (1 nM, HY-10219, MCE) intervention to further study the interaction among Exo-CTCF mediated-IGF2-AS, autophagy, and CDDP resistance in OS cells. TEM ultrastructural analysis showed that after 48 h of CDDP intervention, autophagosomes were increased in MG63 cells treated with Exo-CTCF. After downregulating the expression of IGF2-AS, the regulatory effect of Exo-CTCF on autophagosomes in cells was inhibited. The number of autophagosomes in cells was further increased after rapamycin treatment (Figure 2(g)).

In addition, the expression of p62 in cells cocultured with Exo-CTCF was diminished, and the expression of Beclin1 and the ratio of LC3-II/LC3-I were increased. In comparison with the Exo-CTCF alone, Exo-CTCF and sh-IGF2-AS treatment led to increased expression of p62 and reduced expression of Beclin1 and the ratio of LC3-II/LC3-I. The effects of Exo-CTCF and sh-IGF2-AS treatment were abolished by additional rapamycin treatment (Figure 2(h)). The CCK-8 and flow cytometry showed that cell viability and IC50 value were increased and cell apoptosis was diminished after rapamycin intervention (Figures 2(i) and 2(j)).

The above results indicated that exosomes from CDDP-resistant OS cells transmit CTCF to upregulate the expression of IGF2-AS and activate the autophagy signaling pathway, thereby enhancing the CDDP resistance of OS cells.

### 3.3. IGF2-AS May Competitively Bind to miR-579-3p and Upregulates the Expression of MSH6

**Figure 3:**
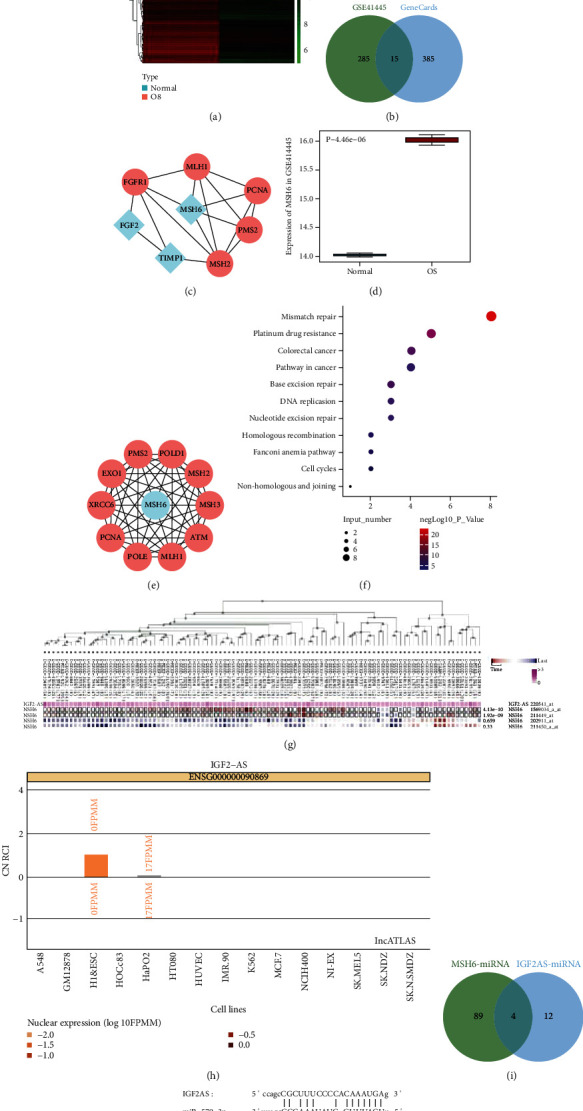
IGF2-AS may competitively bind to miR-579-3p to elevate MSH6 expression in OS. (a) Heat map of top 300 significantly upregulated gene expressions on microarray GSE41445 (normal: *n* = 3; OS: *n* = 3). (b) Venn diagram of the intersection of the top 300 upregulated genes of the microarray GSE41445 and the top 400 OS-associated genes predicted by the GeneCards database. There were 15 genes in the intersection. (c) Interaction network diagram of TIMP1, MSH6, FGF2, and their interaction genes; blue indicated input genes, and red indicated predicted genes. (d) The expression box plot of MSH6 in the microarray GSE41445; the blue box on the left represented the expression of normal samples (*n* = 3), and the red box on the right represented the expression of OS samples (*n* = 3). (e) Interaction network diagram of MSH6 and its interaction genes; blue referred to the input genes, and red referred to the predicted genes. (f) Bubble chart of KEGG enrichment of MSH6 and its interaction genes. The vertical axis represented the enriched item, the horizontal axis and bubble size represented the number of genes enriched in the item, and the color of the bubble indicated significance as shown in the color scale on the right (-log*P* value). (g) Coexpression of IGF2-AS and MSH6 assessed by MEM database analysis. (h) The lncATLAS database analysis to predict the subcellular localization of IGF2-AS. (i) The StarBase database analysis to predict the targeted binding miRNAs of IGF2-AS and MSH6. There were 4 intersecting miRNAs. (j) StarBase database analysis to predict the binding sites between IGF2-AS and miR-579-3p and between miR-579-3p and MSH6. ^∗^*p* < 0.05.

The focus of this study shifted to find the downstream genes of IGF2-AS. The top 300 upregulated genes were retrieved through differential analysis on microarray GSE41445 (Figure 3(a)). The top 400 genes related to OS were obtained from the GeneCards database. Totally, 15 important and significantly upregulated genes were obtained by drawing the Venn diagram based on the intersection of the list of upregulated and OS-related genes (Figure 3(b)). Among them, only TIMP1 [19], MSH6 [20], and FGF2 [21] have been reported in the previous literature to be related to the CDDP resistance of OS cells. The interaction genes of the three genes were predicted using the STRING database, followed by constructing an interaction network. It was found that the core degree of MSH6 was 5, while the core degrees of TIMP1 and FGF2 were both 2 (Figure 3(c)). Therefore, we speculated that MSH6 was one of the most important genes related to OS chemoresistance.

The box plot confirmed that MSH6 was highly expressed in OS (Figure 3(d)). The STRING database was applied again to predict the 10 interaction genes of MSH6 (Figure 3(e)). KOBAS 3.0 was used to perform KEGG enrichment analysis on MSH6 and the interaction genes. It was found that the top 3 KEGG pathways with significant MSH6 enrichment were mismatch repair, CDDP drug resistance, and colorectal cancer (Figure 3(f)). The coexpression prediction of the MEM database found that IGF2-AS and MSH6 were significantly coexpressed (Figure 3(g)). Most of the samples in the significantly coexpressed probes were shown in red, which indicated that their expression was mainly positively correlated.

The lncATLAS database predicted that the subcellular localization of IGF2-AS was in the cytoplasm (Figure 3(h)). IGF2-AS may target MSH6 through miRNAs. Using the StarBase database, it was confirmed that there were 16 and 93 targeted binding miRNAs for IGF2-AS and MSH6, respectively. The intersection of four important miRNAs was miR-4731-5p and miR-579-3p, miR-664b-3p, and miR-3126-5p (Figure 3(i)). Studies have found that the expression of miR-579 was low in OS. Increasing expression of miR-579 could inhibit the development of OS [22]. The StarBase database obtained the binding sites between IGF2-AS and miR-579-3p and between miR-579-3p and MSH6 (Figure 3(j)).

Based on the above results, we speculated that IGF2-AS may competitively bind to miR-579-3p to upregulate the expression of MSH6, thereby enhancing the CDDP resistance of OS cells.

### 3.4. IGF2-AS Competitively Binds to miR-579-3p to Upregulate the Expression of MSH6

**Figure 4:**
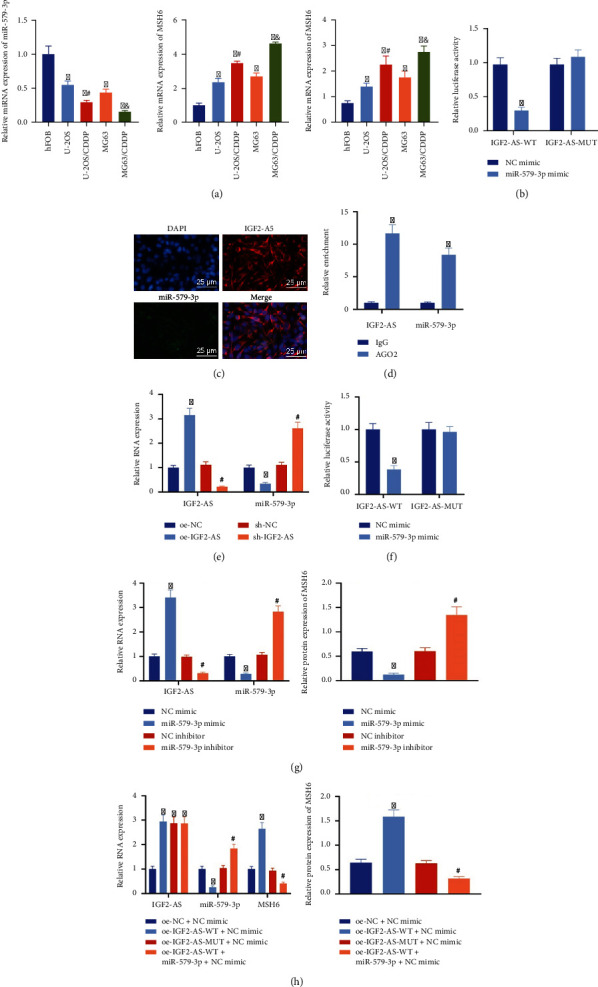
IGF2-AS upregulates the expression of MSH6 by competitively binding to miR-579-3p in OS cells. (a) RT-qPCR and Western blot analysis to determine the expression of miR-579-3p and MSH6 in CDDP-resistant OS cells (^∗^*p* < 0.05*vs.* hFOB cells, ^#^*p* < 0.05*vs.* U-2OS cells, and ^&^*p* < 0.05*vs.* MG63 cells). (b) The dual-luciferase reporter gene assay to verify the binding of IGF2-AS and miR-579-3p. (c) Colocalization of IGF2-AS (red) and miR-579-3p (green) in MG63 cells tested by the FISH assay. The nucleus was stained with DAPI (blue). (d) Detection of the binding of IGF2-AS and miR-579-3p by the RIP assay. (e) The expression of IGF2-AS and miR-579-3p in the cells treated with overexpressed or silenced IGF2-AS measured by RT-qPCR. (f) Binding of miR-579-3p and MSH6 confirmed by the dual-luciferase reporter gene assay. (g) RT-qPCR to determine the expression of IGF2-AS and miR-579-3p and Western blot analysis for MSH6 expression in cells after overexpression or inhibition of miR-579-3p. (h) RT-qPCR to determine the expression of IGF2-AS, miR-579-3p, and MSH6 and Western blot analysis for MSH6 expression in response to oe-IGF2-AS-WT, oe-IGF2-AS-MUT, or oe-IGF2-AS-WT+miR-579-3p mimic. ^∗^*p* < 0.05*vs.* NC mimic/IgG/oe-NC/oe-NC+NC mimic, ^#^*p* < 0.05*vs.* sh-NC/NC inhibitor/oe-IGF2-AS-WT+NC mimic. Cell experiment was repeated three times.

In vitro cell experiments were used to verify the regulatory interaction among IGF2-AS, miR-579-3p, and MSH6. The data of RT-qPCR and Western blot analysis showed that the expression of miR-579-3p was diminished in CDDP-resistant OS cells, and the expression of MSH6 was increased (Figure 4(a)). The dual-luciferase reporter gene assay showed that the miR-579-3p mimic could significantly reduce the luciferase activity of the IGF2-AS-WT plasmid but did not affect the luciferase activity of the IGF2-AS-MUT plasmid, suggesting that IGF2-AS may directly interact with miR-579-3p (Figure 4(b)).

In addition, the FISH assay found that IGF2-AS and miR-579-3p were coexpressed in the cytoplasm (Figure 4(c)). The results of the RIP assay showed that the expressions of IGF2-AS and miR-579-3p in cells treated with AGO2 were higher than those in the cells treated with IgG. This result indicated that there was a direct binding relationship between IGF2-AS and miR-579-3p (Figure 4(d)). IGF2-AS was overexpressed or silenced in MG63 cells, and the RT-qPCR results confirmed that after overexpression of IGF2-AS, the expression of miR-579-3p was diminished, and after silencing the expression of IGF2-AS, the expression of miR-579-3p was increased (Figure 4(e)). The above results indicated that miR-579-3p could be sponged by IGF2-AS.

The dual-luciferase reporter gene assay was used to verify the targeting of miR-579-3p to MSH6. It was found that the miR-579-3p mimic could reduce the luciferase activity of the MSH6-WT plasmid but did not affect MSH6-MUT luciferase activity of the plasmid, which suggested that miR-579-3p may directly interact with MSH6 (Figure 4(f)). Further, overexpression of miR-579-3p reduced the expression of MSH6, and the depleted miR-579-3p elevated the expression of MSH6 (Figure 4(g)). The above results validated that miR-579-3p could target MSH6 and downregulate the expression of MSH6.

In vitro cell experiments were conducted to verify the interaction among IGF2-AS, miR-579-3p, and MSH6. Data of RT-qPCR and Western blot analysis showed that compared with cells treated with oe-NC and NC mimic, the expressions of IGF2-AS and MSH6 were increased, and the expression of miR-579-3p was reduced in cells treated with oe-IGF2-AS-WT and NC mimic. The expression of IGF2-AS was increased in cells with oe-IGF2-AS-MUT and NC mimic treatment, and there was no significant difference in the expression of miR-579-3p and MSH6. In contrast to cells treated with oe-IGF2-AS-WT and NC mimic, the expressions of IGF2-AS and miR-579-3p in cells treated with oe-IGF2-AS-WT and miR-579-3p mimic were increased, while the expression of MSH6 was diminished (Figure 4(h)).

The above results indicated that IGF2-AS could competitively bind to miR-579-3p to upregulate the expression of MSH6 in OS cells.

### 3.5. IGF2-AS/miR-579-3p/MSH6 Axis Activates the Autophagy Signaling Pathway to Enhance CDDP Resistance of OS Cells

**Figure 5:**
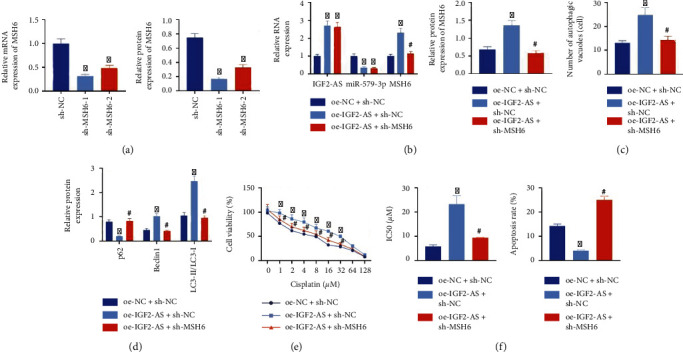
IGF2-AS/miR-579-3p/MSH6 axis activates the autophagy signaling pathway to enhance CDDP resistance of OS cells. (a) RT-qPCR and Western blot analysis for determination of the silencing effect of lentiviral sh-MSH6. (b) Expressions of IGF2-AS, miR-579-3p, and MSH6 in OS cells determined by RT-qPCR and Western blot analysis. (c) The autophagy in the OS cells observed under TEM. (d) Measurement of expressions of autophagy-related genes p62, Beclin1, and LC3 in OS cells by Western blot analysis. (e) The CCK-8 assay to detect the IC50 value of CDDP in OS cells. (f) Flow cytometry analysis to examine the apoptosis of OS cells. ^∗^*p* < 0.05*vs.* sh-NC/oe-NC+sh-NC, ^#^*p* < 0.05*vs.* oe-IGF2-AS+sh-NC. Cell experiment was repeated three times.

We constructed lentiviral sh-MSH6 to further explore whether IGF2-AS conferred its biological function through miR-579-3p/MSH6. The silencing effect of lentiviral sh-MSH6-1 and sh-MSH6-2 was determined, and sh-MSH6-1 was selected for subsequent experiments because of better silencing effect (Figure 5(a)). Besides, overexpression of IGF2-AS increased the expression of MSH6 in MG63 cells, which was reversed by sh-MSH6 (Figure 5(b)).

Ultrastructural analysis of TEM showed that, after CDDP treatment for 48 h, overexpression of IGF2-AS elevated autophagosomes in cells, but it was abolished by silencing of MSH6 (Figure 5(c)). Western blot analysis results manifested that oe-IGF2-AS treatment could decrease the expression of p62 and increase the expression of Beclin1 and the ratio of LC3-II/LC3-I, which were revoked by additional sh-MSH6 treatment (Figure 5(d)). The CCK-8 and flow cytometry showed that cell viability and IC50 value were increased after overexpression of IGF2-AS, and cell apoptosis was diminished, but these effects caused by oe-IGF2-AS could be reversed by sh-MSH6 (Figures 5(e) and 5(f)).

The above results indicated that the IGF2-AS/miR-579-3p/MSH6 axis could activate the autophagy signaling pathway, thereby increasing the resistance of OS cells to CDDP.

### 3.6. Exosomal CTCF Activates the Autophagy Signaling Pathway by Regulating the IGF2-AS/miR-579-3p/MSH6 Axis and Increases the Resistance of OS Cells to CDDP

**Figure 6:**
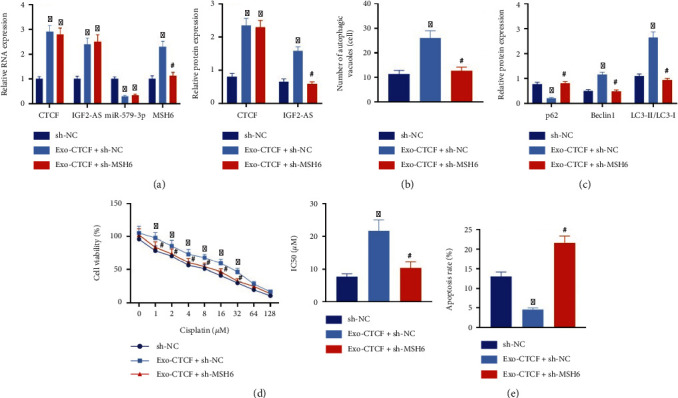
Exosomal CTCF activated the autophagy signaling pathway to facilitate CDDP resistance of OS cells in an IGF2-AS/miR-579-3p/MSH6-dependent manner. (a) RT-qPCR and Western blot analysis for determination of expression of CTCF, IGF2-AS, miR-579-3p, and MSH6 in MG63 cells. (b) Observation of autophagy numbers in MG63 cells under a TEM. (c) Expression of autophagy-related genes p62, Beclin1, and LC3 in MG63 cells measured by Western blot analysis. (d) The CCK-8 assay to evaluate the sensitivity of MG63 cells to CDDP. (e) Apoptosis of MG63 cells assessed by flow cytometry. ^∗^*p* < 0.05*vs.* sh-NC, ^#^*p* < 0.05*vs.* Exo-CTCF+sh-NC. Cell experiment was repeated three times.

Furthermore, exosomes were extracted from MG63/CDDP cells. MG63 cells were cocultured with extracted exosomes and then transduced with lentiviral sh-MSH6. RT-qPCR and Western blot analysis presented that coculture with Exo-CTCF could elevate expression of CTCF, IGF2-AS, and MSH6 and reduce the expression of miR-579-3p, but the effect on MSH6 expression was reversed by sh-MSH6 treatment, accompanied by no significant change on expressions of CTCF, IGF2-AS, and miR-579-3p (Figure 6(a)).

In addition, the ultrastructural analysis of TEM exhibited that Exo-CTCF increased a number of autophagosomes in MG63 cells after CDDP intervention for 48 h, while silencing MSH6 expression reversed the effect of Exo-CTCF caused on the number of autophagosomes in MG63 cells (Figure 6(b)). Western blot analysis results confirmed that Exo-CTCF could diminish p62 level in and elevate expressions of Beclin1 and the ratio of LC3-II/LC3-I, which could be revoked by silencing of MSH6 (Figure 6(c)). The CCK-8 and flow cytometry showed that cell viability and IC50 value were increased and apoptosis was diminished after coculture with Exo-CTCF, but sh-MSH6 could reverse this effect caused by Exo-CTCF (Figures 6(d) and 6(e)).

The above results demonstrated that exosomes from CDDP-resistant OS cells delivered CTCF into OS cells, which activated the autophagy signaling pathway via mediating the IGF2-AS/miR-579-3p/MSH6 axis, thereby increasing the resistance of OS cells to CDDP.

### 3.7. CDDP-Resistant OS Cell-Derived Exosomal CTCF Increases Tumor Formation and CDDP Resistance in Nude Mice Bearing Xenografts of OS Cells

**Figure 7:**
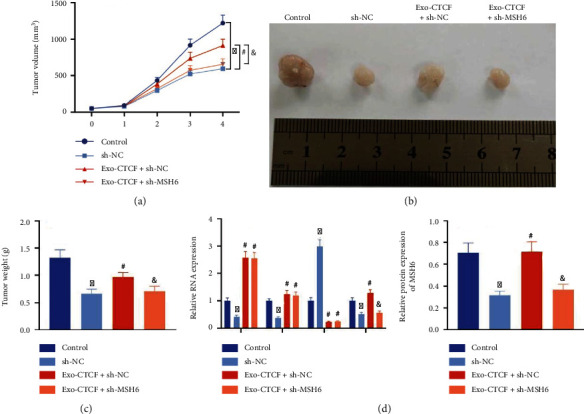
Promoting effect of the exosomal CTCF-mediated IGF2-AS/miR-579-3p/MSH6 axis on tumor formation in nude mice. (a) Tumor volume curves of nude mice. (b) Representative images of subcutaneous xenograft tumors of nude mice. (c) Tumor weight of nude mice. (d) RT-qPCR and Western blot analysis to determine the expressions of CTCF, IGF2-AS, miR-579-3p, and MSH6 in tumors (*n* = 6). ^∗^*p* < 0.05*vs.* control (mice treated with PBS), ^#^*p* < 0.05*vs.* sh-NC or Exo-CTCF+sh-NC.

Next, we investigated the effect of CDDP-resistant OS-Exo-CTCF on the IGF2-AS/miR-579-3p/MSH6 axis by subcutaneous xenograft tumors in nude mice. It was noted that compared with mice with PBS treatment, the weight and volume of tumors in response to CDDP treatment were reduced. Exo-CTCF treatment could promote weight and volume of the tumor, but its effect could be abolished by sh-MSH6 treatment (Figures 7(a)–7(c)).

In addition, the results of RT-qPCR and Western blot analysis presented that the weight and volume of tumors from mice treated with those with CDDP alone were increased in comparison with those with PBS treatment alone. The increased expressions of CTCF, IGF2-AS, and MSH6 and diminished expression of miR-579-3p were caused by Exo-CTCF, and it was confirmed that sh-MSH6 could revoke the impact on MSH6, but it causes no significant effects on expressions of CTCF, IGF2-AS, and miR-579-3p (Figure 7(d)).

The above results validated that CTCF delivered by exosomes derived from CDDP-resistant OS cells could increase the tumorigenesis and CDDP resistance of OS cells in nude mice.

## 4. Discussion

OS is one of the most common primary bone malignancies, and the occurrence of chemotherapy resistance is the main reason for the high incidence of OS patients [23]. CDDP is one of the most widely used agents in the treatment of OS, but the drug resistance of OS to CDDP is a serious problem [24]. Herein, novel therapies tailored with the aid of molecular biomarkers are urgently needed to prevent resistance of OS cells to CDDP. In the present study, we elucidated the regulatory role of CTCF encapsulated by CDDP-resistant OS cell-derived exosomes in CDDP resistance of OS cells via regulation of the IGF2-AS/miR-579-3p/MSH6 axis.

In this study, the OS cell line MG63 was continuously exposed to CDDP to establish CDDP-resistant cell lines (MG63/CDDP). Our findings unraveled higher expression of CTCF in MG63/CDDP cells than in CDDP-sensitive cell lines. Findings obtained from a study confirmed that CTCF is upregulated in colorectal cancer cells and elevated CTCF is able to promote drug resistance in colorectal cancer [25]. Sun et al. also pointed out that knockdown of CTCF can suppress cell progression and chemoresistance in head and neck squamous cell carcinoma [26]. EGR1 and CTCF were found to be involved in the progression of OS cells by interacting with chemokines and their receptors [27]. Similar to these results, our results validated that overexpressed CTCF conferred enhanced CDDP resistance in OS cells. We then validated that CTCF was also overexpressed in exosomes derived from MG63/CDDP cells. It has been reported that CDDP-resistant OS cell-derived exosomes contribute to enhancing chemoresistance of OS cells to CDDP, which could function as a promising biomarker and therapeutic strategy against OS chemoresistance by carrying therapeutic components [28]. As a consequence, it could be concluded that OS cell-derived exosomes transmitted CTCF into OS cells to enhance resistance of OS cells to CDDP. CTCF has been reported to activate downstream gene expression in cancer [29]. Our bioinformatics analysis and tests substantiated that CTCF could bind to the promoter of IGF2-AS as a transcription factor. IGF2-AS has been confirmed to be upregulated and exhibit tumor-promoting properties in various cancers such as hepatocellular carcinoma [30] and breast cancer [31]. Our finding manifested that IGF2-AS was also highly expressed in MG63/CDDP cells. Further evidence revealed that CDDP-resistant cell-derived exosomal CTCF upregulated IGF2-AS to accelerate the autophagy-dependent pathway so as to confer elevated CDDP resistance in OS cells, as companied by elevated autophagosomes and increased Beclin1 expression and the ratio of LC3-II/LC3-I in MG63/CDDP cells. LC3 and Beclin1 are well-known autophagy-related genes and increased the LC3-II/LC3-I ratio, and Beclin1 expression is the hallmark of autophagy [32]. Autophagy is a self-degrading system that is common in the treatment of multidrug resistance in cancers and enables to prevent cancer cells from chemotherapeutic drugs [33]. It has been well documented that autophagy exerts key roles in increasing CDDP resistance in OS cells [34]. By contrary, inhibited autophagy is capable of boosting sensitivity of OS cells to CDDP, along with the reduced Beclin1 level and LC3-II/LC3-I ratio [35, 36]. In recent years, studies have found that lncRNA-mediated dysregulation of targets and pathways is considered to be the key cause of chemotherapy resistance [37, 38]. lncRNAs acting as competitive endogenous RNAs (ceRNAs) have been confirmed through sponging miRNAs to change the expression of their target genes in tumorigenesis [39]. Overexpressed IGF2-AS is confirmed in gastric adenocarcinoma tissues which exhibits a great functional role in promoting cancer cell progression by sponging miR-503 as a ceRNA to regulate SHOX2 expression [12]. Additionally, silencing of IGF2-AS downregulates CREB1 expression through sponging miR-195 to retard tumorigenesis of gastric cancer [40]. Consistent with these findings, the present study validated that IGF2-AS could upregulate MSH6 expression through sponging miR-579-3p to boost resistance of OS cells to CDDP. There is a growing body of evidence supporting the effective role of miR-579-3p played in overcoming drug resistance in cancer treatment [15, 41]. More importantly, miR-579 is underexpressed in OS, and the improvement of miR-579 expression significantly inhibits the development of OS [22]. Moreover, it has been established that MSH6 overexpression promotes the progression of OS [20]. The combined effect of MSH6 gene silencing and CDDP can effectively inhibit the proliferation of OS cells and promote apoptosis [18]. Furthermore, the promoting function of exosomal CTCF-mediated IGF2-AS through the miR-579-3p/MSH6 axis in OS cell resistance to CDDP was confirmed in tumor-bearing nude mice.

**Figure 8:**
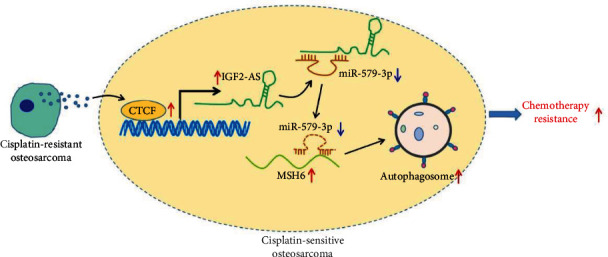
The graphical summary of the function and mechanism of exosomal CTCF in OS. CDDP-resistant OS cell-derived exosomal CTCF activates the IGF2-AS/miR-579-3p/MSH6 axis-mediated autophagy signaling pathway to enhance OS cell resistance to CDDP.

Taken together, the data acquired in this study led to a conclusion that CDDP-resistant OS cell-derived exosomal CTCF activated the autophagy signaling pathway through the IGF2-AS/miR-579-3p/MSH6 axis to enhance OS cell resistance to CDDP (Figure 8). Our findings deepen our understanding of the resistance of OS cells to CDDP and provide a theoretical basis for development of novel targeted therapies to overcome chemotherapy resistance in OS.

## Data Availability

The datasets generated/analyzed during the current study are available within the article.

## Abbreviations

OS: Osteosarcoma
CDDP: Cisplatin
lncRNAs: Long noncoding RNAs
miRNAs: MicroRNAs
miR-579-3p: MicroRNA-579-3p
MSH6: MutS homolog 6
MMR: Mismatch repair
GEO: Gene Expression Omnibus
FBS: Fetal bovine serum
hFOB: Human osteoblasts
HEK-293T: Human embryonic kidney 293T
shRNA: Short hairpin RNA
Exo-CTCF: Effects of exosomes carrying CTCF
CCK-8: Cell Counting Kit-8
PMSF: Phenylmethylsulfonyl fluoride
RIPA: Radioimmunoprecipitation assay
BCA: Bicinchoninic acid
SDS: Sodium dodecyl sulfate
PVDF: Polyvinylidene fluoride
BSA: Bovine serum albumin
TBST: Tris buffered saline with Tween
HRP: Horseradish peroxidase
IgG: Immunoglobulin G
ECL: Electrogenerated chemiluminescence
GAPDH: Glyceraldehyde-3-phosphate dehydrogenase
RT-qPCR: Reverse transcription-quantitative polymerase chain reaction
NTA: Nanoparticle tracking analysis
ChIP: Chromatin immunoprecipitation
WT: Wild type
MUT: Mutant type
RIP: RNA immunoprecipitation
FISH: Fluorescence in situ hybridization
TEM: Transmission electron microscope
EDTA: Ethylenediaminetetraacetate
FITC: Fluorescein isothiocyanate
PI: Propidium iodide
ANOVA: Analysis of variance
ceRNAs: Competitive endogenous RNAs.

## Supplementary Materials

Supplementary 1 Supplementary Figure 1: the IC50 value of OS cells to CDDP by the CCK-8 method, showing the sensitivity of OS cells and CDDP-resistant OS cells; ^∗^*p* < 0.05. Cell experiment was repeated three times. Supplementary Figure 2: identification of the exosomes derived from OS cells and CDDP-resistant OS cells. (A) Observation of exosomes under a TEM. (B) NTA applied to measure the diameter of exosomes. (C) Determination of exosome surface markers by Western blot analysis. Cell experiment was repeated three times.

Supplementary 2 Supplementary Table 1: RT-qPCR primer sequence. Note: RT-qPCR: reverse transcription-quantitative polymerase chain reaction; IGF2-AS: insulin growth factor 2 antisense; CTCF: CCCTC-binding factor; MSH6: MutS homolog 6; GAPDH: glyceraldehyde-3-phosphate dehydrogenase; miR-579-3p: microRNA-579-3p.

Supplementary 3 Supplementary Table 2: query gene IGF2-AS may be regulated by TFs.
