# Supplementary material for: Exosomal CTCF Confers Cisplatin Resistance in Osteosarcoma by Promoting Autophagy via the IGF2-AS/miR-579-3p/MSH6 Axis
Source: J Oncol. 2022 May 31;2022:9390611. doi: 10.1155/2022/9390611 (PMC9175095; doi:10.1155/2022/9390611)
Supplement: Supplementary 1 — Supplementary Figure 1: the IC50 value of OS cells to CDDP by the CCK-8 method, showing the sensitivity of OS cells and CDDP-resistant OS cells; ∗p < 0.05. Cell experiment was repeated three times. Supplementary Figure 2: identification of the exosomes derived from OS cells and CDDP-resistant OS cells. (A) Observation of exosomes under a TEM. (B) NTA applied to measure the diameter of exosomes. (C) Determination of exosome surface markers by Western blot analysis. Cell experiment was repeated three times. [file 9390611.f1.docx]

**Supplementary Figure Legends**

**
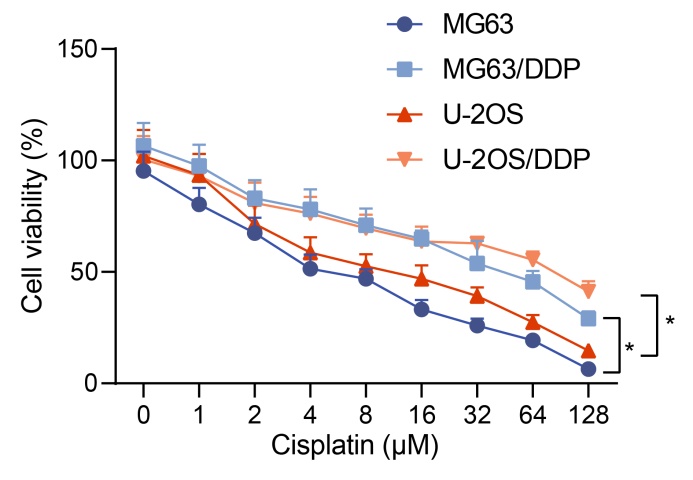
**

**Supplementary Fig. 1** The IC50 value of OS cells to CDDP by CCK-8 method, showing the sensitivity of OS cells and CDDP-resistant OS cells; * *p*< 0.05. Cell experiment was repeated three times.


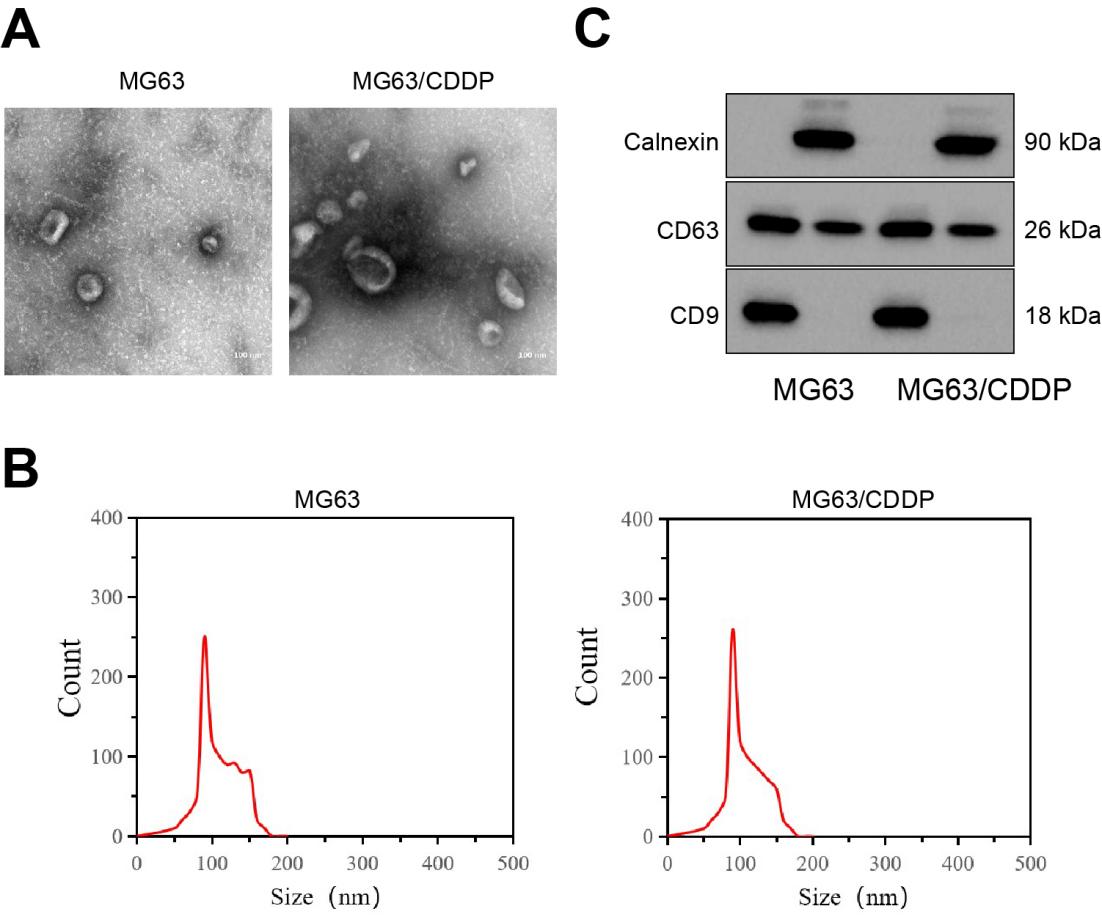


**Supplementary Fig. 2** Identification of the exosomes derived from OS cells and CDDP-resistant OS cells. A, Observation of exosomes under a TEM. B, NTA applied to measure the diameter of exosomes. C, Determination of exosome surface markers by Western blot analysis. Cell experiment was repeated three times.
