# Supplementary material for: Exosomal CTCF Confers Cisplatin Resistance in Osteosarcoma by Promoting Autophagy via the IGF2-AS/miR-579-3p/MSH6 Axis
Source: J Oncol. 2022 May 31;2022:9390611. doi: 10.1155/2022/9390611 (PMC9175095; doi:10.1155/2022/9390611)
Supplement: Supplementary 2 — Supplementary Table 1: RT-qPCR primer sequence. Note: RT-qPCR: reverse transcription-quantitative polymerase chain reaction; IGF2-AS: insulin growth factor 2 antisense; CTCF: CCCTC-binding factor; MSH6: MutS homolog 6; GAPDH: glyceraldehyde-3-phosphate dehydrogenase; miR-579-3p: microRNA-579-3p. [file 9390611.f2.docx]

**Supplementary table 1** RT-qPCR primer sequence

| Gene | Primer sequences（5’-3’） |
| --- | --- |
| IGF2-AS | Forward :CACAAGCTCGGTGGTGACTC |
|  | Reverse :TGAAAGAGGGAGCTCTAGGCA |
| CTCF | Forward :TTCAGGTGGTTAAAGTGGGGGCCAATGGAG |
|  | Reverse :TCCTCTGTATAACGCAGTTTGCTCTTTTTG |
| MSH6 | Forward :CCAAGGCGAAGAACCTCAAC |
|  | Reverse :ACCAGGGGTAACCCTCCATC |
| GAPDH | Forward :ACATCGCTCAGACACCATG |
|  | Reverse :TGTAGTTGAGGTCAATGAAGGG |
| miR-579-3p | Forward :GCGCGTTCATTTGGTATAAACC |
|  | Reverse :AGTGCAGGGTCCGAGGTATT |
| U6 | Forward :CGCTTCGGCAGCACATATACTAA |
|  | Reverse :TATGGAACGCTTCACGAATTTGC |

Note: RT-qPCR, reverse transcription quantitative polymerase chain reaction; IGF2-AS, insulin growth factor 2 antisense; CTCF, CCCTC-binding factor; MSH6, MutS homolog 6; GAPDH, glyceraldehyde-3-phosphate dehydrogenase; miR-579-3p, microRNA-579-3p.
