# Supplementary material for: Exosomal CTCF Confers Cisplatin Resistance in Osteosarcoma by Promoting Autophagy via the IGF2-AS/miR-579-3p/MSH6 Axis
Source: J Oncol. 2022 May 31;2022:9390611. doi: 10.1155/2022/9390611 (PMC9175095; doi:10.1155/2022/9390611)
Supplement: Supplementary 3 — Supplementary Table 2: query gene IGF2-AS may be regulated by TFs. [file 9390611.f3.docx]

Query gene IGF2-AS may be regulated by TFs below. Query gene information: [ ENSG00000099869, antisense, chr11:2140501-2148666 ].

Annotation for the last 2 column: chromesome, peak start, peak end, enrichment socre for the peak, p-value, Qvalue, the distance from the TSS, peak loci (gb:gene body, pr:promoter), which dataset.

Browse peaks No. of datasets TF Tissue No. of peaks (total/average) No. of peaks in gene body (total/average) No. of peaks around TSS (total/average) The peak close to TSS The peak with strongest signal

Details  3 SPI1 Adrenal gland 26/ 8 2 / 0 24 / 8 Chr11,2139131,2139871,9.86,-137,pr,dataset-3155 Chr11,2131502,2132325,12.8,-899,pr,dataset-3155

Details  2 CTCF Aortic adventitial 1/ 0 0 / 0 1 / 0 Chr11,2139225,2139306,5.18,-127,pr,dataset-676 Chr11,2139225,2139306,5.18,-127,pr,dataset-676

Details  1 BCL3 Blood 3/ 3 1 / 1 2 / 2 Chr11,2142948,2143179,17.8,2447,gb,dataset-214 Chr11,2131440,2132154,32.4,-906,pr,dataset-214

Details  2 CBFB Blood 5/ 2 3 / 1 2 / 1 Chr11,2140661,2140934,3.61,160,pr,dataset-333 Chr11,2143876,2144111,5.14,3375,gb,dataset-333

Details  1 CEBPA Blood 1/ 1 1 / 1 0 / 0 Chr11,2142420,2142635,6.85,1919,gb,dataset-5018 Chr11,2142420,2142635,6.85,1919,gb,dataset-5018

Details  1 CEBPB Blood 1/ 1 1 / 1 0 / 0 Chr11,2142398,2142557,5.55,1897,gb,dataset-433 Chr11,2142398,2142557,5.55,1897,gb,dataset-433

Details  69 CTCF Blood 47/ 0 30 / 0 17 / 0 Chr11,2139886,2140032,6.57,-615,pr,dataset-644 Chr11,2143835,2144539,65.3,3334,gb,dataset-4512

Details  1 ELF1 Blood 3/ 3 1 / 1 2 / 2 Chr11,2140691,2140916,5.98,190,pr,dataset-863 Chr11,2140691,2140916,5.98,190,pr,dataset-863

Details  2 EP300 Blood 2/ 1 0 / 0 2 / 1 Chr11,2138756,2138938,6.10,-174,pr,dataset-960 Chr11,2137707,2137838,6.10,-279,pr,dataset-960

Details  3 ERG Blood 11/ 3 7 / 2 4 / 1 Chr11,2140634,2140934,5.39,133,pr,dataset-990 Chr11,2133437,2133751,7.02,-706,pr,dataset-4500

Details  1 FLI1 Blood 4/ 4 4 / 4 0 / 0 Chr11,2146144,2146617,4.82,5643,gb,dataset-1231 Chr11,2146815,2147055,10.2,6314,gb,dataset-1231

Details  1 MAZ Blood 1/ 1 0 / 0 1 / 1 Chr11,2137306,2137556,11.3,-319,pr,dataset-4615 Chr11,2137306,2137556,11.3,-319,pr,dataset-4615

Details  2 PAX5 Blood 7/ 3 4 / 2 3 / 1 Chr11,2139154,2139278,6.17,-134,pr,dataset-2399 Chr11,2148281,2148483,7.71,7780,gb,dataset-2399

Details  2 RAD21 Blood 1/ 0 1 / 0 0 / 0 Chr11,2143896,2144019,6.65,3395,gb,dataset-2603 Chr11,2143896,2144019,6.65,3395,gb,dataset-2603

Details  1 RELA Blood 1/ 1 1 / 1 0 / 0 Chr11,2142908,2143228,8.18,2407,gb,dataset-2713 Chr11,2142908,2143228,8.18,2407,gb,dataset-2713

Details  17 STAG1 Blood 2/ 0 2 / 0 0 / 0 Chr11,2143777,2144062,7.83,3276,gb,dataset-3362 Chr11,2143777,2144062,7.83,3276,gb,dataset-3362

Details  1 STAT1 Blood 2/ 2 2 / 2 0 / 0 Chr11,2142438,2142566,3.12,1937,gb,dataset-3392 Chr11,2142915,2143266,4.31,2414,gb,dataset-3392

Details  1 ESR1 Bone 1/ 1 1 / 1 0 / 0 Chr11,2142978,2143123,4.72,2477,gb,dataset-4080 Chr11,2142978,2143123,4.72,2477,gb,dataset-4080

Details  1 CEBPB Bone marrow 1/ 1 1 / 1 0 / 0 Chr11,2142422,2142569,9.94,1921,gb,dataset-419 Chr11,2142422,2142569,9.94,1921,gb,dataset-419

Details  13 CTCF Bone marrow 4/ 0 4 / 0 0 / 0 Chr11,2143887,2144006,6.41,3386,gb,dataset-738 Chr11,2144263,2144453,10.9,3762,gb,dataset-599

Details  1 EGR1 Bone marrow 2/ 2 0 / 0 2 / 2 Chr11,2139127,2139568,19.0,-137,pr,dataset-841 Chr11,2139127,2139568,19.0,-137,pr,dataset-841

Details  1 MAX Bone marrow 1/ 1 0 / 0 1 / 1 Chr11,2133023,2133132,3.16,-747,pr,dataset-1961 Chr11,2133023,2133132,3.16,-747,pr,dataset-1961

Details  1 MAZ Bone marrow 1/ 1 0 / 0 1 / 1 Chr11,2137294,2137399,6.97,-320,pr,dataset-1990 Chr11,2137294,2137399,6.97,-320,pr,dataset-1990

Details  2 RAD21 Bone marrow 1/ 0 1 / 0 0 / 0 Chr11,2143933,2144021,5.60,3432,gb,dataset-2599 Chr11,2143933,2144021,5.60,3432,gb,dataset-2599

Details  2 SUZ12 Bone marrow 11/ 5 8 / 4 3 / 1 Chr11,2142180,2142375,2.96,1679,gb,dataset-3461 Chr11,2136403,2141731,24.4,-409,pr,dataset-3461

Details  1 ZBTB7A Bone marrow 1/ 1 0 / 0 1 / 1 Chr11,2137318,2137409,5.53,-318,pr,dataset-3796 Chr11,2137318,2137409,5.53,-318,pr,dataset-3796

Details  1 BRD4 Brain 23/ 23 14 / 14 9 / 9 Chr11,2142388,2142648,9.41,1887,gb,dataset-4700 Chr11,2137439,2141457,58.0,-306,pr,dataset-4700

Details  9 CTCF Brain 9/ 1 7 / 0 2 / 0 Chr11,2139219,2139347,8.52,-128,pr,dataset-528 Chr11,2144193,2144552,55.3,3692,gb,dataset-4662

Details  1 EP300 Brain 1/ 1 1 / 1 0 / 0 Chr11,2142920,2143164,10.5,2419,gb,dataset-952 Chr11,2142920,2143164,10.5,2419,gb,dataset-952

Details  1 POLR2A Brain 4/ 4 0 / 0 4 / 4 Chr11,2139089,2139473,12.6,-141,pr,dataset-2532 Chr11,2127619,2130649,31.6,-128,pr,dataset-2532

Details  1 SPI1 Brain 1/ 1 0 / 0 1 / 1 Chr11,2137677,2137911,4.11,-282,pr,dataset-3163 Chr11,2137677,2137911,4.11,-282,pr,dataset-3163

Details  1 TAF1 Brain 2/ 2 0 / 0 2 / 2 Chr11,2139220,2139437,6.67,-128,pr,dataset-3476 Chr11,2137163,2137493,7.43,-333,pr,dataset-3476

Details  1 BRCA1 Breast 2/ 2 0 / 0 2 / 2 Chr11,2131781,2132023,5.37,-872,pr,dataset-251 Chr11,2131781,2132023,5.37,-872,pr,dataset-251

Details  3 BRD4 Breast 2/ 0 1 / 0 1 / 0 Chr11,2139240,2139515,9.03,-126,pr,dataset-5003 Chr11,2139240,2139515,9.03,-126,pr,dataset-5003

Details  5 CEBPB Breast 5/ 1 5 / 1 0 / 0 Chr11,2142247,2142653,62.1,1746,gb,dataset-4973 Chr11,2142264,2142694,63.6,1763,gb,dataset-4971

Details  30 CTCF Breast 16/ 0 16 / 0 0 / 0 Chr11,2143889,2144056,9.25,3388,gb,dataset-699 Chr11,2144223,2144517,15.4,3722,gb,dataset-4003

Details  3 EP300 Breast 3/ 1 3 / 1 0 / 0 Chr11,2142337,2142532,6.27,1836,gb,dataset-4984 Chr11,2142374,2142642,7.83,1873,gb,dataset-4983

Details  16 ESR1 Breast 49/ 3 19 / 1 30 / 1 Chr11,2140749,2140951,4.35,248,pr,dataset-5302 Chr11,2144138,2144522,64.2,3637,gb,dataset-4001

Details  4 FOS Breast 8/ 2 8 / 2 0 / 0 Chr11,2142341,2142611,14.1,1840,gb,dataset-1249 Chr11,2142901,2143200,19.7,2400,gb,dataset-1252

Details  3 FOXA1 Breast 2/ 0 2 / 0 0 / 0 Chr11,2142358,2142572,5.43,1857,gb,dataset-1316 Chr11,2142878,2143183,16.5,2377,gb,dataset-1316

Details  1 HIF1A Breast 4/ 4 0 / 0 4 / 4 Chr11,2139221,2139355,4.08,-128,pr,dataset-1634 Chr11,2137205,2137552,9.76,-329,pr,dataset-1634

Details  7 KDM5B Breast 14/ 2 6 / 0 8 / 1 Chr11,2140995,2141200,3.97,494,pr,dataset-1872 Chr11,2138728,2139513,9.83,-177,pr,dataset-1872

Details  1 NR2F2 Breast 3/ 3 2 / 2 1 / 1 Chr11,2145840,2146249,5.53,5339,gb,dataset-2311 Chr11,2147358,2147619,6.64,6857,gb,dataset-2311

Details  4 SPI1 Breast 21/ 5 14 / 3 7 / 1 Chr11,2142223,2143188,5.86,1722,gb,dataset-3164 Chr11,2137100,2138023,17.9,-340,pr,dataset-3164

Details  2 STAT3 Breast 3/ 1 3 / 1 0 / 0 Chr11,2142410,2142550,5.37,1909,gb,dataset-3401 Chr11,2142973,2143227,12.6,2472,gb,dataset-3405

Details  4 TFAP2C Breast 11/ 2 10 / 2 1 / 0 Chr11,2142451,2142572,6.83,1950,gb,dataset-3595 Chr11,2148358,2148536,10.9,7857,gb,dataset-3601

Details  4 ESR1 Breast Cancer patient-derived xenograft 2/ 0 1 / 0 1 / 0 Chr11,2138913,2139430,31.6,-158,pr,dataset-4063 Chr11,2138913,2139430,31.6,-158,pr,dataset-4063

Details  4 CTCF Cell:LUHMES 8/ 2 5 / 1 3 / 0 Chr11,2139867,2140122,16.5,-634,pr,dataset-4023 Chr11,2139801,2140142,41.3,-700,pr,dataset-4022

Details  1 BRD4 Cell:rhabdomyoblast 7/ 7 7 / 7 0 / 0 Chr11,2142497,2143202,5.15,1996,gb,dataset-4806 Chr11,2144857,2145447,8.47,4356,gb,dataset-4806

Details  1 CTCF Cell:rhabdomyoblast 10/ 10 8 / 8 2 / 2 Chr11,2139752,2140554,12.1,-749,pr,dataset-4810 Chr11,2148253,2149064,95.9,7752,gb,dataset-4810

Details  1 EP300 Cell:rhabdomyoblast 2/ 2 2 / 2 0 / 0 Chr11,2144846,2145246,15.4,4345,gb,dataset-4804 Chr11,2144846,2145246,15.4,4345,gb,dataset-4804

Details  1 CEBPB Cervix 1/ 1 1 / 1 0 / 0 Chr11,2142409,2142563,11.3,1908,gb,dataset-418 Chr11,2142409,2142563,11.3,1908,gb,dataset-418

Details  2 CEBPB Colon 2/ 1 2 / 1 0 / 0 Chr11,2142393,2142532,6.10,1892,gb,dataset-434 Chr11,2142427,2142575,10.6,1926,gb,dataset-435

Details  7 CTCF Colon 11/ 1 7 / 1 4 / 0 Chr11,2139216,2139319,5.36,-128,pr,dataset-764 Chr11,2139211,2139355,11.9,-129,pr,dataset-763

Details  1 EGR1 Colon 1/ 1 0 / 0 1 / 1 Chr11,2139234,2139499,3.86,-126,pr,dataset-843 Chr11,2139234,2139499,3.86,-126,pr,dataset-843

Details  1 POLR2A Colon 2/ 2 0 / 0 2 / 2 Chr11,2139161,2139493,12.4,-134,pr,dataset-2518 Chr11,2139161,2139493,12.4,-134,pr,dataset-2518

Details  4 RAD21 Colon 1/ 0 1 / 0 0 / 0 Chr11,2148381,2148551,4.76,7880,gb,dataset-2609 Chr11,2148381,2148551,4.76,7880,gb,dataset-2609

Details  2 SMC3 Colon 1/ 0 1 / 0 0 / 0 Chr11,2148199,2148511,5.59,7698,gb,dataset-3013 Chr11,2148199,2148511,5.59,7698,gb,dataset-3013

Details  1 SP1 Colon 1/ 1 0 / 0 1 / 1 Chr11,2137761,2138077,3.51,-274,pr,dataset-3066 Chr11,2137761,2138077,3.51,-274,pr,dataset-3066

Details  1 TAF3 Colon 3/ 3 0 / 0 3 / 3 Chr11,2139127,2139302,5.82,-137,pr,dataset-3480 Chr11,2138083,2138330,9.20,-241,pr,dataset-3480

Details  1 JUND Coronary artery 2/ 2 2 / 2 0 / 0 Chr11,2142343,2142636,8.12,1842,gb,dataset-1843 Chr11,2142906,2143209,11.5,2405,gb,dataset-1843

Details  1 FOXA2 Definitive Endoderm 2/ 2 0 / 0 2 / 2 Chr11,2131366,2131747,6.35,-913,pr,dataset-5058 Chr11,2131366,2131747,8.31,-913,pr,dataset-5058

Details  1 CTBP2 Embryo 6/ 6 4 / 4 2 / 2 Chr11,2140632,2140908,4.57,131,pr,dataset-500 Chr11,2137364,2137616,6.20,-313,pr,dataset-500

Details  14 CTCF Embryo 17/ 1 10 / 0 7 / 0 Chr11,2139914,2140034,9.43,-587,pr,dataset-727 Chr11,2139205,2139331,11.4,-129,pr,dataset-541

Details  1 E2F6 Embryo 5/ 5 0 / 0 5 / 5 Chr11,2140022,2140244,11.4,-479,pr,dataset-823 Chr11,2135138,2135304,12.8,-536,pr,dataset-823

Details  4 FOXA2 Embryo 37/ 9 14 / 3 23 / 5 Chr11,2140449,2141335,4.54,-52,pr,dataset-1360 Chr11,2129899,2130504,8.28,-106,pr,dataset-1359

Details  1 FOXP1 Embryo 1/ 1 0 / 0 1 / 1 Chr11,2138091,2138341,5.98,-241,pr,dataset-1400 Chr11,2138091,2138341,5.98,-241,pr,dataset-1400

Details  1 GABPA Embryo 10/ 10 4 / 4 6 / 6 Chr11,2140413,2140914,8.86,-88,pr,dataset-1415 Chr11,2140413,2140914,8.86,-88,pr,dataset-1415

Details  1 MAX Embryo 10/ 10 1 / 1 9 / 9 Chr11,2140748,2141022,3.85,247,pr,dataset-1970 Chr11,2139706,2140211,5.51,-795,pr,dataset-1970

Details  1 PDX1 Embryo 2/ 2 1 / 1 1 / 1 Chr11,2140384,2140539,8.96,-117,pr,dataset-2425 Chr11,2140384,2140539,8.96,-117,pr,dataset-2425

Details  1 POLR2A Embryo 5/ 5 2 / 2 3 / 3 Chr11,2139950,2143418,12.8,-551,pr,dataset-2541 Chr11,2143593,2145337,14.2,3092,gb,dataset-2541

Details  2 RAD21 Embryo 8/ 4 5 / 2 3 / 1 Chr11,2140415,2140593,11.2,-86,pr,dataset-2600 Chr11,2139807,2140111,15.6,-694,pr,dataset-2600

Details  10 SPI1 Embryo 17/ 1 5 / 0 12 / 1 Chr11,2139214,2139428,1.91,-128,pr,dataset-3132 Chr11,2137158,2137749,9.17,-334,pr,dataset-3196

Details  4 SRF Embryo 1/ 0 1 / 0 0 / 0 Chr11,2143942,2144025,4.94,3441,gb,dataset-3348 Chr11,2143942,2144025,4.94,3441,gb,dataset-3348

Details  2 SUZ12 Embryo 11/ 5 5 / 2 6 / 3 Chr11,2140609,2141148,6.90,108,pr,dataset-3454 Chr11,2142012,2145961,33.3,1511,gb,dataset-3453

Details  1 TCF12 Embryo 6/ 6 6 / 6 0 / 0 Chr11,2141032,2141316,5.37,531,gb,dataset-3526 Chr11,2143431,2143660,8.42,2930,gb,dataset-3526

Details  1 GTF2I Foreskin 9/ 9 2 / 2 7 / 7 Chr11,2139704,2140083,6.31,-797,pr,dataset-1557 Chr11,2136967,2137992,8.42,-353,pr,dataset-1557

Details  2 PCGF2 Foreskin 11/ 5 10 / 5 1 / 0 Chr11,2140603,2141822,8.57,102,pr,dataset-2420 Chr11,2143523,2144764,14.8,3022,gb,dataset-2420

Details  5 SPI1 Foreskin 9/ 1 7 / 1 2 / 0 Chr11,2138576,2138932,2.14,-192,pr,dataset-3131 Chr11,2142921,2143150,12.7,2420,gb,dataset-3219

Details  1 ETV1 Gastrointestinal 3/ 3 1 / 1 2 / 2 Chr11,2139247,2139440,9.39,-125,pr,dataset-1170 Chr11,2139247,2139440,9.39,-125,pr,dataset-1170

Details  2 CTCF Gingival 6/ 3 4 / 2 2 / 1 Chr11,2140464,2140573,6.21,-37,pr,dataset-552 Chr11,2140464,2140573,6.21,-37,pr,dataset-552

Details  3 CTCF Heart 3/ 1 3 / 1 0 / 0 Chr11,2143905,2144093,6.39,3404,gb,dataset-691 Chr11,2143905,2144093,6.39,3404,gb,dataset-691

Details  4 SPI1 Heart 9/ 2 1 / 0 8 / 2 Chr11,2139355,2139586,2.70,-114,pr,dataset-3184 Chr11,2137197,2137638,6.65,-330,pr,dataset-3184

Details  1 BRD4 Human Fetal Osteoblasts 3/ 3 0 / 0 3 / 3 Chr11,2138773,2138987,4.78,-172,pr,dataset-4790 Chr11,2137870,2138496,9.11,-263,pr,dataset-4790

Details  1 CEBPB Human Fetal Osteoblasts 1/ 1 1 / 1 0 / 0 Chr11,2142229,2142698,65.5,1728,gb,dataset-4794 Chr11,2142229,2142698,65.5,1728,gb,dataset-4794

Details  2 CTCF Intestinal 6/ 3 5 / 2 1 / 0 Chr11,2139217,2139321,7.82,-128,pr,dataset-522 Chr11,2143888,2144072,10.0,3387,gb,dataset-522

Details  12 SPI1 Intestine 89/ 7 25 / 2 64 / 5 Chr11,2140502,2140702,4.02,1,pr,dataset-3279 Chr11,2137064,2138509,14.6,-343,pr,dataset-3279

Details  6 CTCF Keratinocyte 11/ 1 5 / 0 6 / 1 Chr11,2139205,2139336,9.16,-129,pr,dataset-540 Chr11,2143875,2144062,23.0,3374,gb,dataset-540

Details  1 WDR5 Keratinocyte 14/ 14 3 / 3 11 / 11 Chr11,2140376,2141178,2.82,-125,pr,dataset-3735 Chr11,2137517,2138354,4.54,-298,pr,dataset-3735

Details  2 CTCF Kidney 1/ 0 0 / 0 1 / 0 Chr11,2139186,2139337,9.77,-131,pr,dataset-695 Chr11,2139186,2139337,9.77,-131,pr,dataset-695

Details  2 EP300 Kidney 4/ 2 4 / 2 0 / 0 Chr11,2142653,2143231,10.2,2152,gb,dataset-4914 Chr11,2142653,2143231,10.2,2152,gb,dataset-4914

Details  1 RYBP Kidney 8/ 8 4 / 4 4 / 4 Chr11,2140333,2141508,7.60,-168,pr,dataset-2881 Chr11,2143699,2144794,8.85,3198,gb,dataset-2881

Details  11 SPI1 Kidney 22/ 2 1 / 0 21 / 1 Chr11,2140350,2140594,1.48,-151,pr,dataset-3134 Chr11,2137197,2137651,5.83,-330,pr,dataset-3229

Details  1 ZNF263 Kidney 2/ 2 0 / 0 2 / 2 Chr11,2136880,2138383,15.1,-362,pr,dataset-3833 Chr11,2136880,2138383,15.1,-362,pr,dataset-3833

Details  1 CEBPB Liver 2/ 2 1 / 1 1 / 1 Chr11,2139245,2139455,5.11,-125,pr,dataset-421 Chr11,2139245,2139455,5.11,-125,pr,dataset-421

Details  4 CREB1 Liver 23/ 5 0 / 0 23 / 5 Chr11,2139171,2139420,16.4,-133,pr,dataset-4388 Chr11,2139171,2139420,16.4,-133,pr,dataset-4388

Details  8 CTCF Liver 25/ 3 16 / 2 9 / 1 Chr11,2140453,2140604,11.7,-48,pr,dataset-575 Chr11,2143852,2144076,27.8,3351,gb,dataset-575

Details  1 ELF1 Liver 4/ 4 1 / 1 3 / 3 Chr11,2139233,2139429,5.27,-126,pr,dataset-857 Chr11,2136735,2136963,5.86,-376,pr,dataset-857

Details  1 FOSL2 Liver 10/ 10 1 / 1 9 / 9 Chr11,2142432,2142587,6.73,1931,gb,dataset-1267 Chr11,2128122,2130721,18.5,-123,pr,dataset-1267

Details  2 FOXA1 Liver 12/ 6 0 / 0 12 / 6 Chr11,2139192,2139468,4.65,-130,pr,dataset-1274 Chr11,2128031,2130756,27.5,-124,pr,dataset-1274

Details  2 FOXA2 Liver 15/ 7 1 / 0 14 / 7 Chr11,2140390,2140963,8.14,-111,pr,dataset-5056 Chr11,2133467,2133820,11.7,-703,pr,dataset-5056

Details  1 GABPA Liver 1/ 1 0 / 0 1 / 1 Chr11,2131915,2132090,3.49,-858,pr,dataset-4387 Chr11,2131915,2132090,3.49,-858,pr,dataset-4387

Details  1 HNF4A Liver 4/ 4 0 / 0 4 / 4 Chr11,2139212,2139485,5.99,-128,pr,dataset-1649 Chr11,2132356,2132557,9.09,-814,pr,dataset-1649

Details  1 JUND Liver 12/ 12 1 / 1 11 / 11 Chr11,2139138,2139480,16.8,-136,pr,dataset-1824 Chr11,2128113,2130568,20.5,-123,pr,dataset-1824

Details  2 MAX Liver 18/ 9 2 / 1 16 / 8 Chr11,2139783,2140201,6.44,-718,pr,dataset-1972 Chr11,2139114,2139472,7.54,-138,pr,dataset-1972

Details  1 MAZ Liver 3/ 3 0 / 0 3 / 3 Chr11,2139826,2139991,5.08,-675,pr,dataset-1989 Chr11,2137250,2137624,11.3,-325,pr,dataset-1989

Details  2 MYC Liver 2/ 1 0 / 0 2 / 1 Chr11,2139906,2140006,6.02,-595,pr,dataset-2138 Chr11,2139906,2140006,6.02,-595,pr,dataset-2138

Details  1 NR2F2 Liver 12/ 12 0 / 0 12 / 12 Chr11,2139138,2139281,3.28,-136,pr,dataset-2309 Chr11,2128557,2130527,4.42,-119,pr,dataset-2309

Details  4 POLR2A Liver 22/ 5 0 / 0 22 / 5 Chr11,2139737,2139993,6.55,-764,pr,dataset-2472 Chr11,2128051,2132470,78.6,-124,pr,dataset-2472

Details  3 RAD21 Liver 10/ 3 5 / 1 5 / 1 Chr11,2140414,2140588,10.5,-87,pr,dataset-2601 Chr11,2143800,2144082,46.0,3299,gb,dataset-2601

Details  1 REST Liver 6/ 6 0 / 0 6 / 6 Chr11,2139182,2139475,10.1,-131,pr,dataset-2760 Chr11,2139182,2139475,10.1,-131,pr,dataset-2760

Details  1 SIN3A Liver 12/ 12 0 / 0 12 / 12 Chr11,2139915,2140063,4.62,-586,pr,dataset-2902 Chr11,2139200,2139441,9.59,-130,pr,dataset-2902

Details  1 SP1 Liver 1/ 1 0 / 0 1 / 1 Chr11,2139365,2139501,5.90,-113,pr,dataset-3059 Chr11,2139365,2139501,5.90,-113,pr,dataset-3059

Details  1 STAG1 Liver 5/ 5 3 / 3 2 / 2 Chr11,2140463,2140590,6.19,-38,pr,dataset-3354 Chr11,2143814,2144078,38.7,3313,gb,dataset-3354

Details  1 TAF1 Liver 4/ 4 0 / 0 4 / 4 Chr11,2139144,2139484,5.36,-135,pr,dataset-3472 Chr11,2137209,2137365,5.50,-329,pr,dataset-3472

Details  1 YY1 Liver 20/ 20 3 / 3 17 / 17 Chr11,2140328,2140583,7.46,-173,pr,dataset-3765 Chr11,2139112,2139489,12.0,-138,pr,dataset-3765

Details  1 ZBTB7A Liver 12/ 12 1 / 1 11 / 11 Chr11,2140352,2140568,14.8,-149,pr,dataset-3797 Chr11,2140352,2140568,14.8,-149,pr,dataset-3797

Details  2 BRCA1 Lung 12/ 6 5 / 2 7 / 3 Chr11,2141099,2141583,4.45,598,gb,dataset-4953 Chr11,2131317,2132041,6.73,-918,pr,dataset-4953

Details  3 CEBPB Lung 3/ 1 3 / 1 0 / 0 Chr11,2142301,2142642,45.5,1800,gb,dataset-423 Chr11,2142301,2142642,45.5,1800,gb,dataset-423

Details  20 CTCF Lung 13/ 0 9 / 0 4 / 0 Chr11,2139923,2140051,7.65,-578,pr,dataset-573 Chr11,2139164,2139333,12.9,-133,pr,dataset-598

Details  1 EP300 Lung 1/ 1 0 / 0 1 / 1 Chr11,2137756,2137959,4.18,-274,pr,dataset-968 Chr11,2137756,2137959,4.18,-274,pr,dataset-968

Details  1 FOSL2 Lung 1/ 1 1 / 1 0 / 0 Chr11,2142361,2142589,4.71,1860,gb,dataset-1268 Chr11,2142361,2142589,4.71,1860,gb,dataset-1268

Details  1 JUND Lung 1/ 1 1 / 1 0 / 0 Chr11,2142471,2142574,6.38,1970,gb,dataset-1829 Chr11,2142471,2142574,6.38,1970,gb,dataset-1829

Details  1 LMNB1 Lung 13/ 13 4 / 4 9 / 9 Chr11,2140262,2140532,2.67,-239,pr,dataset-1931 Chr11,2143826,2144915,3.50,3325,gb,dataset-1931

Details  1 MAX Lung 2/ 2 0 / 0 2 / 2 Chr11,2139847,2139970,3.99,-654,pr,dataset-1971 Chr11,2139210,2139340,4.81,-129,pr,dataset-1971

Details  1 MAZ Lung 2/ 2 0 / 0 2 / 2 Chr11,2138564,2138688,3.49,-193,pr,dataset-1991 Chr11,2137216,2137517,8.66,-328,pr,dataset-1991

Details  1 MYC Lung 1/ 1 0 / 0 1 / 1 Chr11,2139187,2140159,13.3,-131,pr,dataset-2115 Chr11,2139187,2140159,13.3,-131,pr,dataset-2115

Details  2 POLR2A Lung 5/ 2 0 / 0 5 / 2 Chr11,2139240,2139429,7.01,-126,pr,dataset-2507 Chr11,2139145,2139462,11.1,-135,pr,dataset-2506

Details  3 RAD21 Lung 2/ 0 2 / 0 0 / 0 Chr11,2143847,2144054,7.52,3346,gb,dataset-2612 Chr11,2143847,2144054,7.52,3346,gb,dataset-2612

Details  1 RELA Lung 5/ 5 5 / 5 0 / 0 Chr11,2142414,2142595,11.4,1913,gb,dataset-2727 Chr11,2142922,2143206,29.7,2421,gb,dataset-2727

Details  1 SP1 Lung 2/ 2 0 / 0 2 / 2 Chr11,2137666,2137896,4.40,-283,pr,dataset-3061 Chr11,2137266,2137583,4.83,-323,pr,dataset-3061

Details  14 SPI1 Lung 39/ 2 7 / 0 32 / 2 Chr11,2139126,2139356,2.56,-137,pr,dataset-3282 Chr11,2137184,2137632,8.26,-331,pr,dataset-3254

Details  1 CTCF Lung/bronchus 2/ 2 2 / 2 0 / 0 Chr11,2143873,2144015,8.68,3372,gb,dataset-721 Chr11,2143873,2144015,8.68,3372,gb,dataset-721

Details  1 STAT3 Lymphocyte 3/ 3 1 / 1 2 / 2 Chr11,2142990,2143218,6.29,2489,gb,dataset-3412 Chr11,2134522,2134664,8.35,-597,pr,dataset-3412

Details  1 FLI1 Mesenchymal 2/ 2 0 / 0 2 / 2 Chr11,2136968,2137255,3.81,-353,pr,dataset-1234 Chr11,2130924,2131116,3.81,-957,pr,dataset-1234

Details  47 SPI1 Muscle 237/ 5 100 / 2 137 / 2 Chr11,2140483,2140696,3.62,-18,pr,dataset-3118 Chr11,2137034,2137993,31.6,-346,pr,dataset-3161

Details  2 MYOD1 Myotube 2/ 1 2 / 1 0 / 0 Chr11,2144817,2145156,26.1,4316,gb,dataset-2176 Chr11,2144817,2145156,26.1,4316,gb,dataset-2176

Details  1 BRD4 Nervous system 5/ 5 0 / 0 5 / 5 Chr11,2139928,2140074,4.75,-573,pr,dataset-293 Chr11,2139128,2139481,8.09,-137,pr,dataset-293

Details  1 KDM4C Oesophagus 2/ 2 1 / 1 1 / 1 Chr11,2141148,2141319,4.28,647,gb,dataset-1863 Chr11,2137090,2137263,6.12,-341,pr,dataset-1863

Details  1 CEBPB Other 1/ 1 1 / 1 0 / 0 Chr11,2142465,2142570,4.09,1964,gb,dataset-437 Chr11,2142465,2142570,4.09,1964,gb,dataset-437

Details  12 CTCF Other 14/ 1 10 / 0 4 / 0 Chr11,2140434,2140577,13.7,-67,pr,dataset-751 Chr11,2143858,2144073,14.4,3357,gb,dataset-572

Details  1 DDX5 Other 2/ 2 0 / 0 2 / 2 Chr11,2139139,2139711,5.66,-136,pr,dataset-790 Chr11,2131406,2132174,7.50,-909,pr,dataset-790

Details  1 DUX4 Other 1/ 1 0 / 0 1 / 1 Chr11,2130344,2130521,6.10,-101,pr,dataset-799 Chr11,2130344,2130521,6.10,-101,pr,dataset-799

Details  1 KLF9 Other 2/ 2 0 / 0 2 / 2 Chr11,2139070,2139389,6.40,-143,pr,dataset-1904 Chr11,2137247,2138294,11.0,-325,pr,dataset-1904

Details  8 SPI1 Other 34/ 4 12 / 1 22 / 2 Chr11,2140723,2140926,1.47,222,pr,dataset-3299 Chr11,2138445,2139496,7.72,-205,pr,dataset-3090

Details  6 CTCF Pancreas 3/ 0 2 / 0 1 / 0 Chr11,2139148,2139393,6.49,-135,pr,dataset-626 Chr11,2143792,2144176,25.4,3291,gb,dataset-626

Details  6 SPI1 Penis 19/ 3 8 / 1 11 / 1 Chr11,2139190,2139536,3.44,-131,pr,dataset-3272 Chr11,2137124,2137694,10.3,-337,pr,dataset-3212

Details  5 SPI1 Placenta 25/ 5 5 / 1 20 / 4 Chr11,2140856,2141156,3.99,355,pr,dataset-3120 Chr11,2137119,2137904,13.3,-338,pr,dataset-3138

Details  1 CEBPA Pleura 1/ 1 1 / 1 0 / 0 Chr11,2142426,2142554,8.34,1925,gb,dataset-411 Chr11,2142426,2142554,8.34,1925,gb,dataset-411

Details  1 MAZ Primitive interlobular tissue 29/ 29 11 / 11 18 / 18 Chr11,2140304,2140534,5.40,-197,pr,dataset-4174 Chr11,2135735,2140170,46.0,-476,pr,dataset-4174

Details  5 AR Prostate 8/ 1 4 / 0 4 / 0 Chr11,2142324,2142775,85.1,1823,gb,dataset-3878 Chr11,2142324,2142775,85.1,1823,gb,dataset-3878

Details  1 CBX8 Prostate 1/ 1 0 / 0 1 / 1 Chr11,2136902,2137452,5.86,-359,pr,dataset-351 Chr11,2136902,2137452,5.86,-359,pr,dataset-351

Details  8 CTCF Prostate 12/ 1 7 / 0 5 / 0 Chr11,2139919,2140090,6.74,-582,pr,dataset-712 Chr11,2143855,2144127,22.2,3354,gb,dataset-712

Details  1 EZH2 Prostate 12/ 12 5 / 5 7 / 7 Chr11,2140108,2140636,5.86,-393,pr,dataset-4916 Chr11,2138235,2138829,13.7,-226,pr,dataset-4916

Details  1 SUMO2/SUMO3 Prostate 2/ 2 2 / 2 0 / 0 Chr11,2143944,2144117,4.91,3443,gb,dataset-3447 Chr11,2143944,2144117,4.91,3443,gb,dataset-3447

Details  1 SUZ12 Prostate 17/ 17 9 / 9 8 / 8 Chr11,2140715,2141218,17.3,214,pr,dataset-3458 Chr11,2140130,2140663,19.8,-371,pr,dataset-3458

Details  1 EP300 Renal cortex 15/ 15 6 / 6 9 / 9 Chr11,2140507,2143257,12.6,6,pr,dataset-3895 Chr11,2138445,2139299,18.6,-205,pr,dataset-3895

Details  8 SPI1 Renal cortex 13/ 1 1 / 0 12 / 1 Chr11,2139271,2139492,2.33,-123,pr,dataset-3119 Chr11,2137223,2137837,4.31,-327,pr,dataset-3113

Details  11 SPI1 Renal pelvis 18/ 1 0 / 0 18 / 1 Chr11,2139297,2139541,2.47,-120,pr,dataset-3107 Chr11,2137193,2137697,5.51,-330,pr,dataset-3105

Details  4 BRD4 Retina 32/ 8 12 / 3 20 / 5 Chr11,2140557,2140888,3.62,56,pr,dataset-4934 Chr11,2136947,2138111,27.1,-355,pr,dataset-4931

Details  3 CTCF Retina 4/ 1 1 / 0 3 / 1 Chr11,2139734,2140025,4.35,-767,pr,dataset-4936 Chr11,2139065,2139432,17.6,-143,pr,dataset-4936

Details  16 CTCF Skin 3/ 0 2 / 0 1 / 0 Chr11,2139224,2139336,9.57,-127,pr,dataset-549 Chr11,2139224,2139336,9.57,-127,pr,dataset-549

Details  20 SPI1 Skin 35/ 1 21 / 1 14 / 0 Chr11,2142352,2142611,3.49,1851,gb,dataset-3303 Chr11,2142895,2143236,9.53,2394,gb,dataset-3192

Details  2 CTCF Spinal cord 7/ 3 3 / 1 4 / 2 Chr11,2140461,2140557,6.20,-40,pr,dataset-563 Chr11,2139224,2139332,6.89,-127,pr,dataset-563

Details  3 SPI1 Spinal cord 7/ 2 2 / 0 5 / 1 Chr11,2140481,2140943,5.25,-20,pr,dataset-3172 Chr11,2137218,2137909,6.93,-328,pr,dataset-3172

Details  9 SPI1 Stomach 74/ 8 30 / 3 44 / 4 Chr11,2140490,2141145,5.29,-11,pr,dataset-3217 Chr11,2137250,2138085,15.4,-325,pr,dataset-3266

Details  1 SPI1 Testes 2/ 2 0 / 0 2 / 2 Chr11,2140039,2140239,2.78,-462,pr,dataset-3153 Chr11,2137199,2137604,4.27,-330,pr,dataset-3153

Details  2 SPI1 Umbilical cord blood 10/ 5 3 / 1 7 / 3 Chr11,2139053,2139432,3.31,-144,pr,dataset-3247 Chr11,2138511,2138873,3.67,-199,pr,dataset-3247

Details  1 EGR1 Uterus 1/ 1 0 / 0 1 / 1 Chr11,2139224,2139553,12.0,-127,pr,dataset-844 Chr11,2139224,2139553,12.0,-127,pr,dataset-844

Details  1 NFIC Uterus 1/ 1 0 / 0 1 / 1 Chr11,2139353,2139559,8.35,-114,pr,dataset-2243 Chr11,2139353,2139559,8.35,-114,pr,dataset-2243

Details  1 RAD21 Uterus 1/ 1 1 / 1 0 / 0 Chr11,2143901,2144007,5.30,3400,gb,dataset-2611 Chr11,2143901,2144007,5.30,3400,gb,dataset-2611

Details  1 USF1 Uterus 2/ 2 0 / 0 2 / 2 Chr11,2134456,2134748,5.33,-604,pr,dataset-3712 Chr11,2132949,2133285,10.6,-755,pr,dataset-3712

Details  1 ZBTB7A Uterus 8/ 8 1 / 1 7 / 7 Chr11,2140388,2140518,4.18,-113,pr,dataset-3798 Chr11,2137397,2138102,9.03,-310,pr,dataset-3798

Details  8 CTCF Vein 6/ 0 2 / 0 4 / 0 Chr11,2140453,2140577,7.58,-48,pr,dataset-705 Chr11,2139176,2139305,9.33,-132,pr,dataset-705

Details  1 ETS1 Vein 1/ 1 1 / 1 0 / 0 Chr11,2144287,2144459,4.88,3786,gb,dataset-1155 Chr11,2144287,2144459,4.88,3786,gb,dataset-1155

Details  1 FLI1 Vein 3/ 3 0 / 0 3 / 3 Chr11,2137803,2138056,3.25,-269,pr,dataset-1235 Chr11,2136806,2136980,4.11,-369,pr,dataset-1235

Details  1 RELA Vein 1/ 1 1 / 1 0 / 0 Chr11,2142886,2143263,12.5,2385,gb,dataset-2737 Chr11,2142886,2143263,12.5,2385,gb,dataset-2737
